# MapIO: A Gestural and Conversational Interface for Tactile Maps

**DOI:** 10.1109/access.2025.3566286

**Published:** 2025-05-01

**Authors:** MATTEO MANZONI, SERGIO MASCETTI, DRAGAN AHMETOVIC, RYAN CRABB, JAMES M. COUGHLAN

**Affiliations:** 1 Department of Computer Science, Università degli Studi di Milano, 2133 Milan, Italy; 2 The Smith-Kettlewell Eye Research Institute, San Francisco, CA 94115, USA

**Keywords:** Assistive technologies, blind and low vision people, conversational interface, digitally augmented tactile maps, tactile maps

## Abstract

For individuals who are blind or have low vision, tactile maps provide essential spatial information but are limited in the amount of data they can convey. Digitally augmented tactile maps enhance these capabilities with audio feedback, thereby combining the tactile feedback provided by the map with an audio description of the touched elements. In this context, we explore an embodied interaction paradigm to augment tactile maps with conversational interaction based on Large Language Models, thus enabling users to obtain answers to arbitrary questions regarding the map. We analyze the types of questions the users are interested in asking, engineer the Large Language Model’s prompt to provide reliable answers, and study the resulting system with a set of 10 participants, evaluating how the users interact with the system, its usability, and user experience.

## I. INTRODUCTION

Tactile maps are essential tools for Blind and Low Vision (BLV) people, providing spatial information through raised lines, symbols, and braille. They are relatively inexpensive, and commercial services such as the LightHouse’s Tactile Maps Automated Production^1^ make it possible to order a map of any desired region (*e.g*., the neighborhood of a given address), which dramatically expands access to tactile maps beyond the limited set of generic ones (*e.g*., world, country and state) traditionally available in schools [1]. Alternatively, it is straightforward for a teacher or Orientation & Mobility instructor to create simple tactile maps, for example, using a tool such as Sensational Blackboard^2^ to inscribe streets and other map features with only pen and paper. This expanded availability of tactile maps allows a BLV individual to learn about any region. This learning may be for educational purposes or can enable the individual to acquaint themselves with a region in advance of traveling there, thereby increasing their familiarity with the region and confidence navigating it in person.

Tactile maps, however, can convey only a limited amount of information due to space constraints and the nature of tactile encoding. Digitally Augmented Tactile Maps (DATMs) mitigate this limitation by providing additional information associated to the specific point of the map that the user is exploring through audio feedback. This form of interaction through *point inspection* enhances the amount and richness of spatial and contextual information that can be presented to the user.

While DATMs overcome the information amount limit of traditional tactile maps, they still have a major flaw. Indeed, while an unlimited amount of information can be associated to a given point on the map, accessing a great quantity of data provided as audio feedback is time consuming and cognitively demanding for the user. Thus, only so much information can be provided, and a critical design choice becomes which information to provide. This problem is compounded by the fact that retrieving information of interest may require the user to traverse a multitude of points on the map. For example, to know what restaurants are open at 9pm in a given area, a user would need to traverse all points of interest in the area and listen to their audio feedback until information about their opening time is provided.

To address this problem, this paper investigates a new form of DATM that combines *point inspection* with *verbal queries* that the user can ask to the system to receive a verbal answer. The proposed solution, called *MapIO*, leverages Large Language Models (LLMs) to answer these queries by also using lexical models. augmenting them with additional *query context*, that includes the point on the map the user is touching, relevant map details not inherently known to the LLM (*e.g*., points of interest on the map), and guidelines provided to the LLM to improve the answer quality and reduce the risk of hallucinations. This solution was designed to allow the model to encode a large amount of information, while, at the same time, allowing the user to effectively access this information selectively as needed.

During the process of designing *MapIO*, we addressed the following research questions:

**RQ1** What kinds of questions would BLV people ask to a DATM that can be accessed through *verbal queries*?
**RQ2** Can these questions be reliably answered by an existing LLM model?
  **RQ2.1** How to design LLM *query context* in order to achieve accurate answers?
**RQ3** How would users interact with such a system?
**RQ4** How users perceive such a system and its functionalities in terms of usability, usefulness, and clarity?

**FIGURE 1.**
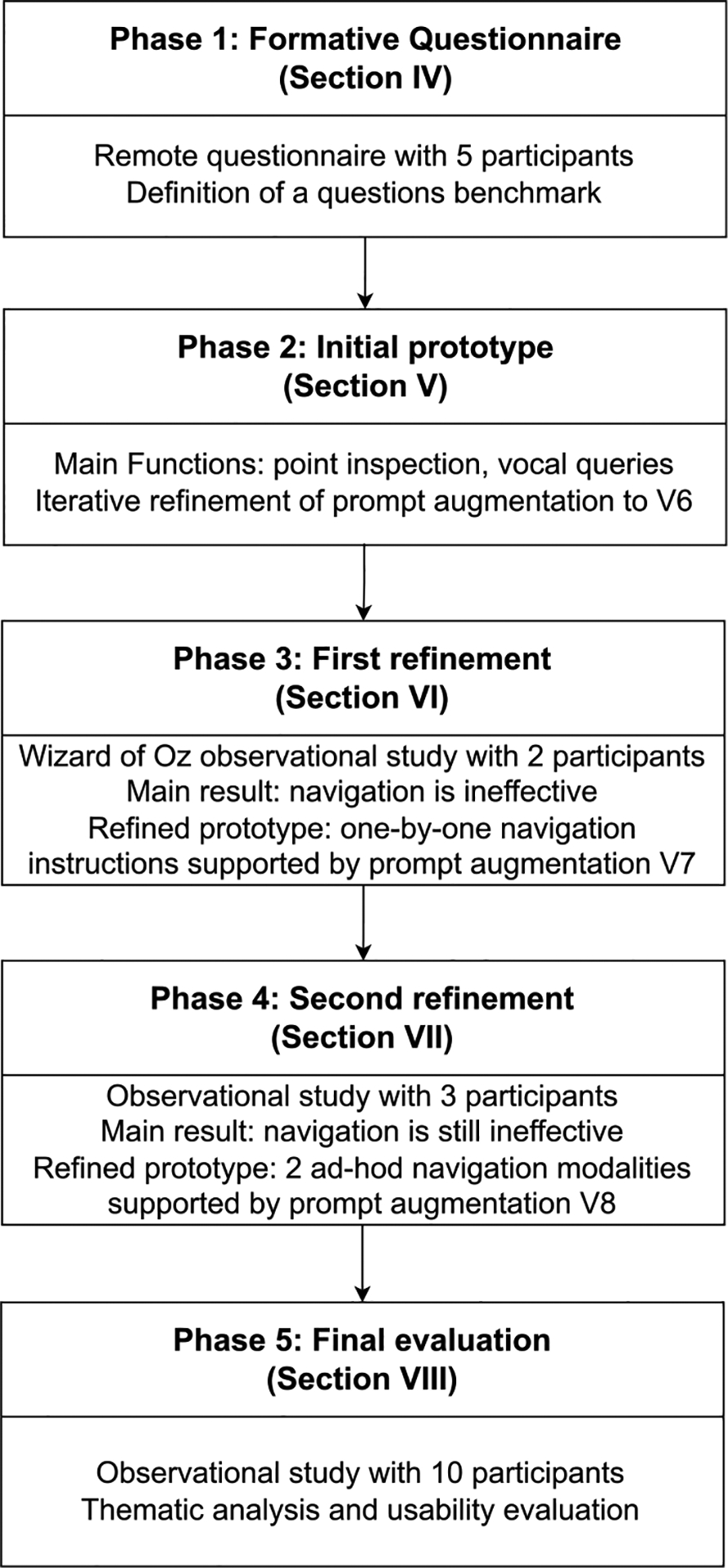
Phases in *MapIO* development.

To answer these research questions, we follow a usercentric, iterative design approach, structured in five phases, as depicted in Figure 1. In the **first phase** (Section IV), through a formative study conducted with 5 BLV participants and teachers of BLV students, we identified the goals, tasks, and types of questions that BLV people have when using tactile maps (**RQ1**). In particular, we collected a set of benchmark questions that the participants would ask to a DATM augmented with conversational interaction capabilities.

In the **second phase** (Section V), based on the outcomes of the formative study, we designed an initial prototype of *MapIO*, a system that supports *point inspection* and *verbal queries*. Prompt augmentations and query context were then iteratively refined until satisfactory results were achieved, measured as the truthfulness and completeness of the LLM’s responses to the benchmark questions (**RQ2**).

In the **third phase** (Section VI), we conducted an observational study with 2 participants during which the conversational interaction is simulated by a supervisor who provides answers by reading LLM responses, correcting obvious errors. Interaction with the system was effective through both *point inspection* and *verbal queries*. However, the answers to requests for directions included all navigation instructions needed to reach the destination, making them too long and hard to remember. Thus, we refined *MapIO* to provide these navigation instructions one-by-one.

In the **fourth phase** (Section VII) we conducted a new study with 3 participants and a complete version of *MapIO*, observing that, while the system functioned properly and separated navigation instructions were easier to follow, the navigation was still ineffective because it required the user to ask multiple queries, each of which took a few seconds to process. Hence, we created a second refinement of *MapIO*, adding two ad-hoc navigation modalities to guide the user to the destination without the need to ask multiple queries.

In the **final phase** (Section VIII), we conducted a user study with 10 participants. Through visual observations, we analyzed how participants interact with *MapIO* and, in particular, how they manage errors, incomplete information, and uncertainty during their interactions (**RQ3**). Moreover, through a final interview and questionnaires we assessed subjective feedback regarding the system, considering aspects such as usefulness, usability and clarity (**RQ4**).

Results show that users are interested in learning about points of interest, their properties (*e.g*., opening hours), road accessibility, and directions (**RQ1**). While existing LLMs have limitations in spatial reasoning [2], prompt engineering, combined with ad-hoc functions, can enhance their performance to near-perfect results (**RQ2**). Effective prompts should incorporate a structured representation of the road network, including nodes, edges, and accessibility data (*e.g*., type of paving) alongside details of points of interest like location and operating hours (**RQ2.1**). The study also sheds light on how BLV users interact with the system and in particular that they effectively alternate *point inspection* with *verbal queries* despite some difficulties, in particular related to the *navigation* function, that we identified and discuss later in the paper (**RQ3**). Finally, system functionalities were deemed to be useful and clear, and the system usability was rated positively (**RQ4**).

## II. RELATED WORK

Tactile materials are indispensable tools for BLV people to access information [3]. Previous literature has addressed the problem of digitally improving the accessibility of tactile materials. This body of research is described in Section II-A. Our work contributes to this research domain through the design of a novel embodied interaction paradigm for the accessibility of tactile maps that combines manual exploration and conversational querying using LLMs. To this end, we also survey prior research in the field of assistive technologies based on the use of conversational interfaces, in particular for the accessibility of tactile materials (see Section II-B).

### A. DIGITALLY AUGMENTED TACTILE MATERIALS

Tactile materials can be 2D or 3D. A common approach to the creation of 2D tactile materials is to use embossed paper which can be created with special printers or manually drawn with special tools like the Sensational Blackboard^2^. Instead, 3D models can be made with 3D printers [4]. In all these cases the result is a physical model.

An alternative solution to generate 2D tactile representations is to use a refreshable tactile display, a computer peripheral that contains an array of tactile pins [5]. Since the height of each pin can be controlled though a computer program, these devices can generate refreshable tactile images, including tactile maps [6]. Refreshable tactile displays are an emerging technology that promises to revolutionize the availability of tactile data far beyond what is possible with conventional physical materials and indeed this approach is adopted in patents [7], [8] and commercial solutions, including Graphiti,^3^ Monarch,^4^ and the Feelif Pro Tablet.^5^ One characteristic that is particularly relevant is that these devices can display dynamic information, a feature that can enable new forms of interactions, for example it can be used to allow scrolling and zooming into a tactile map [9]. On the other hand, these devices are expensive, so they have limited adoption. Their spatial resolution is also limited, in particular compared with traditional paper-based maps. For these reasons, the system presented in this paper was designed to work with physical maps. However, the technique proposed in this paper can be easily extended to augment a digital map represented on a refreshable tactile display.

A known challenge with tactile materials is to balance the need to represent a large amount of information while maintaining readability. In the case of tactile maps, for example it would be useful to report information such as road names, accessibility features, and points of interest. On the other side, representing all such information in braille or raised text is infeasible in most cases. To address this problem, several papers in the literature have proposed to digitally augment the tactile materials to provide additional information through audio or vibrations.

Digital augmentation can be achieved with two sensing techniques: touch-based and vision-based. In touch-based solutions, a common approach used for 2D materials is to position a tactile paper over a touchpad (such as a touchscreen, for example) so that the user can explore the tactile paper while simultaneously allowing the detection of touches on the touchpad. T3 Tactile Tablet^6^ is a commercial solution using this approach, while [10] patents a similar system which can be used to access information printed on physical documents. Brock et al. have also used this technique to show that augmenting tactile materials with audio feedback can improve their accessibility [11]. The same technique was also specifically used to augment tactile maps [12], [13], [14], [15]. Conductive elements inserted into 3D printed tactile maps [16] or 3D models [17] can also be used. This allows the user to touch pre-determined points of the model to trigger audio feedback. Finally, refreshable tactile displays with touch sensing can also be used to provide audio feedback related to the touched elements [9].

In vision-based digital augmentation, a camera is used to detect when the user is pointing to an element of a tactile material, hence triggering audio feedback. For 2D models, this solution was adopted using standard RGB cameras [18], [19] or RGBD (color plus depth) cameras [20]. Tactile Images^7^ is a commercial product using this approach that supports the exploration of tactile maps and other types of tactile materials by tracking the user’s finger position and providing audio feedback about the pointed area. The same approach can be used for 3D models, again using RGB cameras [21], [22], or RGBD ones [23], [24].

One challenge of the computer vision approach is to interpret user intent, in particular to understand which part of the model the user is pointing to. For this, some systems require a specific pointing tool [22], while others ask users to augment their fingertip with a visual marker [21]. These methods, however, are often perceived as unnatural [22], [25] and may not support simultaneous interactions with multiple hands or fingers. With the rise of advanced hand-tracking algorithms, touch-based interfaces are becoming more natural, intuitive and versatile [18], [20], [24].

Existing solutions, regardless of how they are implemented (via touch or computer vision), all use *point inspection*. This form of interaction mitigates the problem of encoding a large amount of information into the model itself but it does not completely solve it, as motivated in the Introduction. The solution presented in this paper addresses this problem by combining *point inspection*, which can provide quick access to a limited set of information, with *verbal queries* that gives the user access to a much larger set of information.

### B. USE OF CONVERSATIONAL INTERFACES IN ASSISTIVE TECHNOLOGIES

Conversational interfaces have previously been proposed in the literature to improve the accessibility of tactile materials. For example, *LucentMaps* [16] enables interaction with tactile maps through touch and speech. Similarly, the *Jido* project [26] supports interaction with 2D tactile maps and adds real-world navigation to specific points of interest using a companion app. Despite the benefits of these conversational interfaces, both *Jido* and *LucentMaps* support only a limited set of queries, which restricts their capacity for comprehensive interaction. Examples of supported queries in *Jido* include “Is there a <point of interest> around?”, “What is this?”, and “Guide me to <point of interest>.” The solution proposed in this paper, instead, adopts a conversational interfaces that supports a larger set of queries thanks to the use of a LLM.

Large Language Models have also been previously used in the broader field of assistive technologies. For example, EasyAsk is an in-app contextual tutorial search assistant designed to simplify app usage on smartphones through voice and touch inputs [27]. This system leverages LLMs to process spoken requests and contextual information, enhancing accessibility for older adults. ChartLama [28] and VizAbility [29] are LLM-based systems that enhance chart accessibility by allowing users to ask questions about chart content. However, ChartLama is limited to charts generated through a specific pipeline, whereas VizAbility can autonomously interpret chart content using Olli’s tree view [30], making it easier to integrate into existing screen readers. Similarly, Cuadra et al. [31] used an LLM-based system to improve accessibility in “health data entry” forms. Their system combines LLMs and multimodal interfaces to allow users, including those with disabilities, to complete complex forms independently. Finally, another recent work [32] explores through a Wizard-of-Oz study how a refreshable tactile display could be augmented with LLM capabilities to verbally answer to user questions regarding a displayed data visualization, while also dynamically updating the tactile representation accordingly.

In the navigation field, NaviGPT [33] is a mobile application designed to enhance travel experiences for BLV individuals. Combining obstacle detection, vibration feedback, and an LLM-based conversational interface, NaviGPT provides continuous feedback through image recognition and contextual navigation. The LLM processes nearby landmarks, routes, and the user’s current position, as provided by Apple Maps, to answer user queries. Finally, Tran et al. [34] utilize an LLM to support BLV individuals who are learning to use tactile maps. A key distinction between Tran’s system and *MapIO* is that Tran’s system operates solely with digital maps of very simple indoor environments, while *MapIO* aims to support more complex outdoor environments. Additionally, the LLM in Tran’s system can answer user queries but lacks knowledge of the user’s position on the map and cannot assist with navigation tasks.

## III. METHODOLOGY

Our iterative design methodology relies on a series of user studies to inform system interaction and prompt augmentation refinements. User studies conducted in Phases 3, 4 and 5 (see Figure 1) all follow a similar methodology, which is described in Section III-A. Analogously, Phases 2, 3 and 4 adopt the same methodology for the iterative refinement of the prompt augmentation, which is described in Section III-B.

### A. USER STUDIES

The evaluation of *MapIO* is conducted through observational studies, followed by interviews with participants. The study was approved by the ethics committee of The Smith-Kettlewell Eye Research Institute (Approval #: COU001) and we obtained informed content from each participant.

#### 1) MEASUREMENTS AND ANALYSES

During observational studies we investigated how participants interact with the system (**RQ3**). To this end, we acquired video and audio recordings using a camera that frames the map and the participant’s hands. We also collected additional system logs and debug information, including conversational interaction transcriptions and information regarding the computer vision functions (*e.g*., whether the hands are in the pointing position and the coordinates pointed).

The interview data was used to assess participants’ subjective feedback regarding system usefulness, usability, and clarity of its functionalities (**RQ4**). During Refinement Phases (Sections VI and VII), due to a small number of participants, we conducted an informal analysis of this data. Instead, in the Final Evaluation, we analyzed the data using reflexive thematic analysis [35] and System Usability Scale (SUS) [36].

#### 2) APPARATUS, SETTING, AND STIMULI

The study was conducted in a lab at The Smith-Kettlewell Eye Research Institute, with the participant sitting at a table in a quiet room. A tactile map and an Apple MacBook Pro M2 laptop running *MapIO* were positioned in front of the participant. An external camera was connected to the laptop and mounted above the tactile map to track the map and the participant’s hands. We used two maps: one of the Empire State Building neighborhood in Manhattan, New York City, NY, USA, and another of the Conant Gardens neighborhood in Detroit, Michigan, USA. Some points of interest on both maps were fictional and specifically created for these experiments. The New York map was used for training, while the Detroit map was used for the actual tasks. The maps were purchased from the LightHouse for the Blind and Visually Impaired^1^.

#### 3) EXPERIMENTAL PROTOCOL

The study was organized into three stages. In the first stage, we collected participants’ demographic information including age, gender, visual condition, self-reported expertise with braille, tactile maps, and LLMs (on a Likert-like scale “None”, “Low”, “Mid”, “High”), whether they were teachers of BLV students, and whether they had previous experience with digitally augmented tactile materials. We then provided a brief introduction to the study, outlined the system’s functionalities, and encouraged participants to ask questions at any point and to adopt a think-aloud approach throughout the study (although we also suggested participants not talk to the supervisor while asking verbal questions to the LLM).

The second stage was the training, during which we gradually introduced participants to the system using the New York map. We first explained how to use *point inspection* and asked them to complete basic tasks, such as identifying a road and obtaining additional details about it. Next, we demonstrated how to interact through *verbal queries* and prompted participants to ask questions themselves, such as inquiring about restaurants on the map.

The third stage was the formal evaluation, during which each participant was asked to complete ten tasks using the Detroit map. Tasks included actions like exploring the map, inquiring the system for specific points of interest, and locating them on the map. The complete list of tasks is available in the Supplementary Material. Each task had a set time limit, after which the supervisor would proceed to the next task. During this phase, the supervisor was not permitted to assist participants in task completion. However, if the user failed to complete a task and a subsequent task depended on its successful completion, the supervisor showed the participant how to complete it.

### B. PROMPT AUGMENTATION ENGINEERING

The purpose of prompt augmentation engineering is to improve the quality of the LLM’s responses (**RQ2**) by refining the *query context* (**RQ2.1**). We first describe the query answering data flow (Section III-B1) and then the methodology we adopted to improve the quality of the LLM’s responses (Section III-B2).

#### 1) QUERY ANSWERING DATA FLOW

**FIGURE 2.**
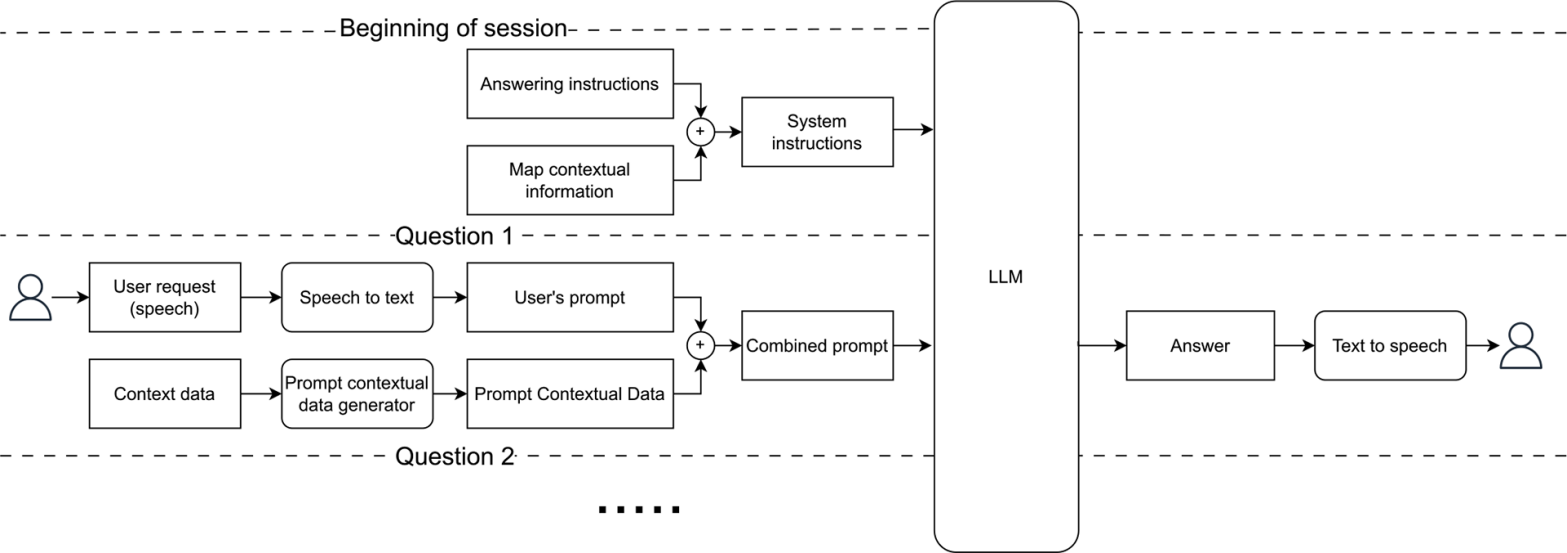
Data flow involving the LLM with focus on prompt augmentation.

Figure 2 depicts the query answering data flow. The *query context*, which we aim to refine, consists of *system instructions* and *prompt contextual data*.

**System instructions** are text directives that can be provided to the LLM at the beginning of each session to guide the model’s behavior.^8^ These are available in most modern LLMs (including ChatGPT and Gemini). We organize system instructions into *answering instructions* and *map contextual information*. The **map contextual information** consists of a description of the map, the area where it is located, possibly together with additional information, like a road graph, points of interest, etc. This information provides context for the conversational interaction which would otherwise be unknown to the LLM. The **answering instructions**, instead, include a set of directives to specify the lexical context (e.g., “I’m a blind person who needs help to navigate a new neighborhood.”), and set the tone for the conversation (e.g., “Ensure that your answer is unbiased and does not rely on stereotypes.”). Additionally, the answering instructions define which tool calls the LLM can use. Tool calls are a feature offered by the LLM model we used^9^ that allows it to call local functions to complete specific tasks. Each tool call includes a description of the provided functionality and the explanation of when the LLM should call it. When the LLM decides to use a tool call it returns a specific request that contains the name of the function to execute and its parameters. *MapIO* then executes the function and sends the result back to the LLM, which ultimately produces the final answer to the user’s query.

Every time the user asks a verbal question, a speech-to-text module converts it into text (the *user’s prompt*) and concatenates it with the **prompt contextual data** that describes the context specific to a given user’s prompt, such as the position indicated on the map at the time of the request. Prompt contextual data are generated from the contextual data by an algorithm called the *prompt contextual data generator*. The result of the concatenation of *user’s prompt* and *prompt contextual data* is the *combined prompt*, which is provided to the LLM. Finally, the LLM answer is read aloud to the user.

#### 2) PROMPT AUGMENTATION ENGINEERING METHODOLOGY

To improve the quality of the answers, we iteratively refine the answering instructions, the map contextual information and the prompt contextual data generator. The procedure uses on a benchmark of 38 user questions, each associated with a user prompt, a context, and the expected answer. An example of a request is: “Tell me the roads parallel to this one”, the context is a position pointed on the map which correspond to a given road, and the answer contains the names of the two roads that are parallel to the one that is pointed to. The questions were designed based on the formative questionnaire (see Section IV) and follow the classification into landmark, route, and survey queries previously proposed in the literature [11]. The complete list of questions is provided as Supplementary Material.

The process was iterative. In each iteration, we defined the prompt augmentation technique as a combination of the system instructions and a prompt contextual data generator. We then provided to the LLM the system instructions and, for each request in the benchmark, the combined prompt, obtained by combining the user’s prompt with the prompt contextual data generated from the context using the specified prompt contextual data generator. Through a manual process, this response is evaluated against the expected response and classified into the following categories: Deceptively wrong, Not replying to question, Blatantly wrong, Partial or incomplete, Correct but not optimal, Correct.

At each iteration we assigned an incremental version number to the prompt augmentation technique. This methodology makes it possible to compare the different versions by measuring the results in objective terms.

## IV. PHASE 1: FORMATIVE QUESTIONNAIRE

We conducted a formative study to comprehend how BLV people could use an ideal DATM integrated with conversational interaction capabilities, and to collect questions that users would ask to such a system. The study was organized as a remote questionnaire with BLV participants and teachers of BLV students. Specifically, the questionnaire describes an ideal DATM that is capable of tracking the point of the map touched by the user, reading the names of streets and intersections, and that can answer general questions asked verbally, which can also refer to the current position pointed by the user on the map. Through two open ended questions, we ask the participants to illustrate what questions they would ask the system and for what purposes they would use it. The complete questionnaire is reported in Supplementary Material.

The questionnaire was administered via email to four BLV adults. Specifically, **P1**, **P2** and **P3** are blind individuals, while **P4** has low vision. Three of the them are also teachers of BLV students (**P1**, **P3**, and **P4**). One additional participant, **P5**, is not BLV and has experience as a teacher of BLV students. Table 1 summarizes participants’ demographic data.

All participants expressed an interest in receiving general information about the map, with requests such as *“Give me an overall description of the map”* (**P1**) or *“What can I find on the map?”* (**P3**). Note that these requests would not need to refer to a specific location on the map. A similar question is *“How many streets are there?”* (**P2**). The participants also suggested questions that are related to a specific position on the map. Specifically, **P1**, **P2**, **P3**, and **P4** expressed interest in knowing the position they are currently pointing on the map, while **P1**, **P2**, **P3**, and **P5** also wanted a detailed description of the location. For example, **P1** was interested in knowing what they could find along the road that they are touching on the map, while **P2** expressed interest for more general details about streets. Accessibility information was mentioned by both **P4** and **P5**, with requests like *“Tell me if there are stairs at some point on the road”* (**P4**).

All participants suggested using the DATM to familiarize themselves with an unfamiliar environment and explore its road network and points of interest before actually visiting. **P1** and **P2** also indicated they would use the system to find specific locations on the map, such as *“a street, a school, and so on”* (**P2**). Additionally, **P1**, **P2**, and **P5** mentioned that they would use the system to get directions to reach certain places. **P1** noted they would ask *“How can I come here from …?”*, while **P4** mentioned they would use the system *“to explore a route and decide whether I can go on foot or by taxi or other public transportation.”*. **P5** also stated they would use the system to teach BLV individuals how *“to understand a complex intersection, traffic patterns, route planning, etc.”*.

Ultimately, based on the participants’ answers, we curated a list of benchmark questions (**RQ1**) that our system should be capable of addressing (reported in Supplementary Material). The benchmark questions were used for prompt augmentation engineering (Section III-B).

## V. PHASE 2: INITIAL PROTOTYPE

Motivated by the limitations with the existing DATM approaches (see Section II-A), and the use cases reported in the formative study (see Section IV), we designed a novel DATM system, called *MapIO*. The system is designed to enable embodied interaction with a tactile map, providing tactile feedback (from the physical map) as well as audio feedback through *point inspection* and *verbal queries*. These queries also consider contextual information related to the map and the knowledge of the element touched by the user.

### A. SYSTEM AND INTERACTION DESIGN

The interaction through *point inspection* requires the user to point to elements on the map with the index finger. Upon pointing, *MapIO* reads the name of the element pointed to, and the reading stops if the finger moves away from that element. This natural interaction is similar to prior approaches [11], [19], [21], [22], [23], [25], in particular those using computer vision-based hand tracking [18], [20], [24]. When no pointing hand is found, the system plays a background sound of crickets chirping. Thus, the tactile maps can be freely explored with open hands without triggering the reading of the touched elements.

The conversational interface allows the user to verbally request information regarding streets, intersections, and points of interest. For streets, this information includes the paving material, slope (if any), traffic direction (if one-way street), and accessibility features or barriers (*e.g*., presence of tactile pavings). For intersections, it includes the type of the intersection (*e.g*., a “T” intersection), presence of crosswalks or traffic lights, and accessibility features (*e.g*., crosswalk audio signal). For points of interest, the features may include opening times, facilities (*e.g*., Wi-Fi) and accessibility features (*e.g*., whether an elevator is available).

To ask questions, the user presses the space bar on the keyboard and begins speaking. When the space bar is pressed again, the recording of the question stops and the captured audio is converted into text using speech-to-text. A notification sound indicates the start and the end of recording. Optionally, the user can point at an element on the map while asking the question, so that the pointed position is used as context data when generating the prompt contextual data. The text returned by the LLM is then read aloud using text-to-speech and a chime sound is played at the end of the message. The user can also halt long answers to *verbal queries* by pressing the *Enter* key on the keyboard.

### B. SYSTEM IMPLEMENTATION

**FIGURE 3.**
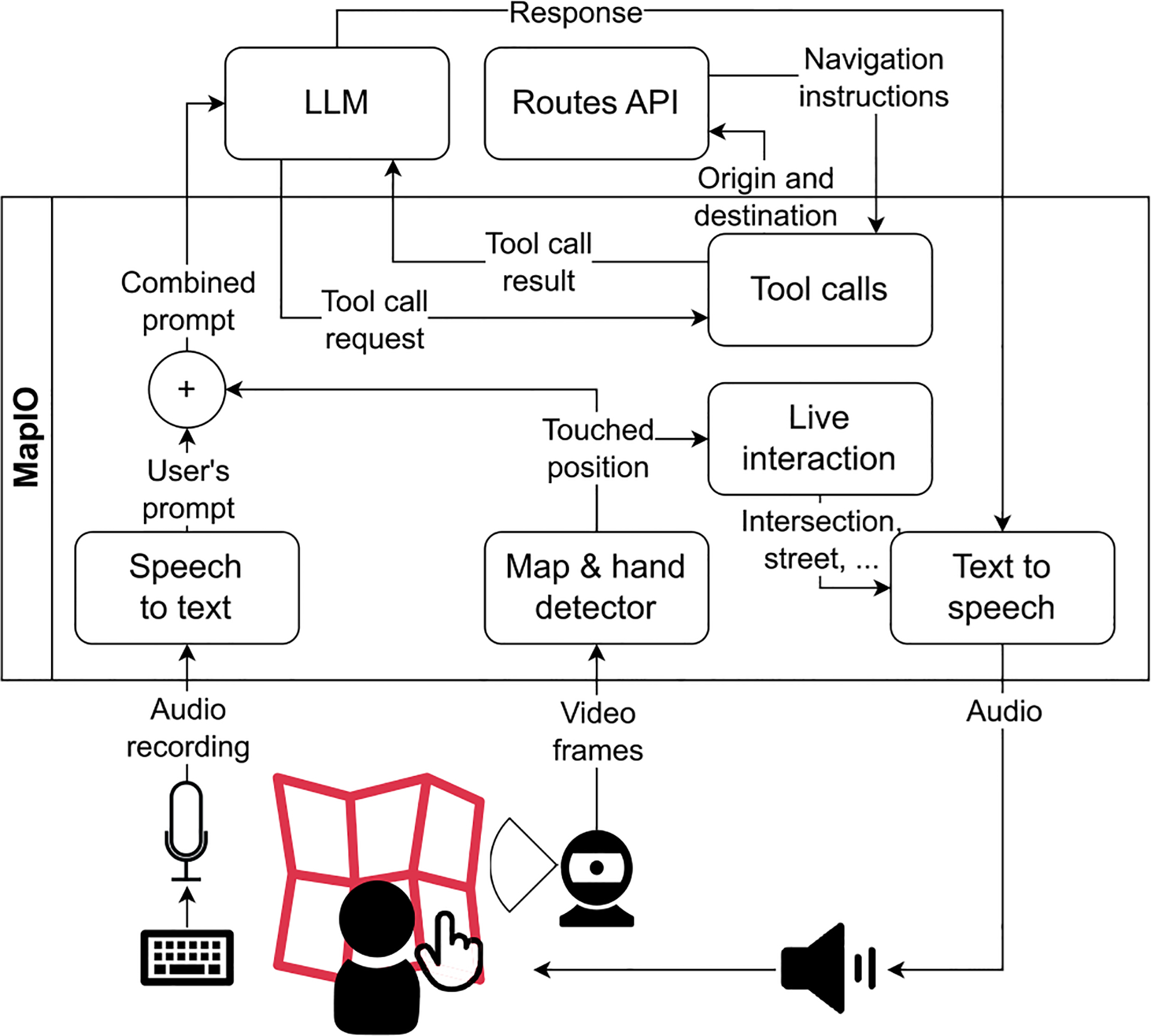
*MapIO* initial design architecture.

*MapIO* is implemented as a computer application within the architecture depicted in Fig. 3. A video camera is used to acquire a video stream of a tactile map placed on a desk, and of the hands of a user interacting with the map, at approximately 10 frames per second. The images are processed by the *Map & hand detector* module, that detects the map by matching SIFT features [37] of a map image template (previously stored by *MapIO*), and those extracted from video frames. The same module computes a homography matrix to convert the coordinates from the frame reference system to that of the map. Finally, the *Map & hand detector* module tracks the hands and checks if there is a pointing gesture. This is achieved by using the MediaPipe library [38] that produces a list of 20 landmarks for each hand. If the landmarks of the index finger are all aligned, and those of the other fingers are not, a pointing gesture is detected (thumb landmarks are ignored). The pointing position corresponds to the landmark of the tip of the index finger, converted to the map reference system. To reduce tracking noise, the detected index positions are averaged over a 10-frame window.

To enable natural interaction with the map, *MapIO* also has a map model containing a graph of the streets and intersections displayed on the map, points of interest present in the area, and additional information about those elements, such as opening hours, facilities, and accessibility features or barriers. Through computer speakers, the *Live interaction* module provides audio feedback on the elements pointed to by the user. These can be sound cues, or verbal messages synthesized using a text-to-speech engine.

When the user asks a *verbal query*, the recording is converted into text by the Google API speech-to-text module.^10^ The text transcription is then forwarded to a LLM^9^ along with the pointed to position and relevant information about the map model, as described in Section III-B. The answer provided by the LLM is played using text-to-speech.^11^

Note that *point inspection* provides almost instantaneous feedback. By contrast, the total time to compute an answer to a *verbal query* can vary from a fraction of a second to more than ten seconds. During this time, the system does not provide audio feedback through *point inspection*. Instead, every 7 seconds, a verbal message informs the user that the system is computing the answer.

### C. PROMPT AUGMENTATION

We iteratively explored different versions of the prompt augmentation (see Table 2). We now briefly describe the first five versions and then describe the sixth version, used in the first *MapIO* prototype. For a detailed description of the first five versions, see the Supplementary Material.

The first version relied mostly on pre-existing LLM knowledge to generate responses, while the contextual information contained only a basic description of the map area (*e.g*.: “We are in New York, in the Empire State Building area”) and the user’s address (*e.g*.: “I am at 51 West 36th Street”). This resulted in 50% of correct responses. In the attempt to improve the answer accuracy, in the second and third versions we relied on the LLM’s abilities to process multimodal input, providing a context image in addition to a textual prompt. In the second version we provided a picture of the map and the user’s hands taken from the camera, while in the third version we provided a synthetic image of the map containing a marker indicating the touched point. Both approaches decreased the performance compared to Version 1. We therefore abandoned the idea of providing multimodal input to the LLM and only provided geometric information extracted from the camera image, which includes the coordinate and address of the touched point. This was augmented with a description of the road graph (in JSON format in Version 4, and in text form in the following versions). This approach actually increased the percentage of correct answers to 81.5% in Version 5. Despite the improved performance, the LLM kept struggling with questions that require advanced spatial reasoning. For example, in question 8 of the benchmark (“Will I make it to <POI> before it closes?”), it incorrectly responded that it was possible to reach the destination before it closed, despite the user being positioned very far away from it and having only 5 minutes to reach it.

As a solution to the LLM’s poor spatial reasoning abilities [2], in the sixth version we leverage the support for tool calls implemented in OpenAI’s GPT-4o to complete specific spatial reasoning tasks with ad-hoc procedures. Specifically, we use tool calls to compute the distance between two points (or between a point and a point of interest), to answer whether a given position is close to a given point of interest, to return a list of points of interest close to a given point, and to list instructions to navigate between two points (or between the pointed location and a point of interest). For this last functionality, we intercept requests for directions from the user and forward them to Google Routes API^12^ that, given a starting point and a destination, would return street-by-street instructions on how to navigate between the two. Thanks to the use of these tool calls, we increased the number of correct responses to 89.47%, with only a single response classified as blatantly wrong and no deceptively wrong answers.

## VI. PHASE 3: FIRST REFINEMENT

### A. EVALUATION

We evaluated the initial prototype with 2 participants, one blind (**P6**) and one with low vision (**P7**) (see Table 3). Both had prior experience as teachers of BLV students, and both are proficient with braille and tactile maps. They have also previously participated in studies using digitally augmented tactile materials.

The evaluation adopted the methodology described Section III-A, with the only difference that we wanted to give the experimenter the chance to correct blatantly wrong LLM answers. Hence, we deactivated the text-to-speech reading of the LLM output. Instead, an experimenter would read the text answers provided by the LLM, correcting them if needed (similar to a Wizard-of-Oz approach).

Both participants effectively used the system, taking advantage of a combination of the three exploration modes: tactile, *point inspection*, and *verbal queries*. In particular, we observed that the participants used both hands to explore the map, often pointing with one hand to activate *point inspection*, while exploring with the other open hand. In some cases *point inspection* was indispensable, for example to disambiguate braille labels in the vicinity of multiple streets. Similarly, *verbal queries* were used to address problems that could not be otherwise solved, like acquiring information about points of interest and locating them on the map. No major errors occurred in the LLM answers. Thus, the experimenter did not have to correct the LLM output. This underlines the effectiveness of the designed prompt augmentation technique.

Despite these encouraging results, we also noticed four issues. First, *MapIO* incurred occasional errors when the hands were located outside the map area. Second, by comparing the recorded video with log information, we observed that in some cases the detected pointed position was inaccurate. This caused a few problems, including the fact that, when the user was pointing to an intersection, nearby road names were erroneously announced. The third problem was related to LLM answers when the user asks for directions to reach a given point of interest. The main problem was that the LLM answer included the complete list of directions, which participants had difficulties to memorize. Consequently, the participants would coarsely explore the area referring to whatever they remembered of the instructions. Another problem, reported by **P6**, was that there was no way to know if the target point of interest was reached if not by asking the system, which requires an additional verbal interaction. Finally, the instructions were perceived to be overly verbose as well, since they contained also accessibility instructions regarding each traversed street and intersection. On the other hand, the provided instructions did not include the distance, which was deemed useful by both users (**P7** explicitly commented about this, while **P6** queried the system for this information). The final issue was that *MapIO* was unable to distinguish between 4-way and T intersections, while we observed that this information could be useful for the user.

### B. REFINED PROTOTYPE

To address the limitations emerged during the first iteration, we applied the following modifications to the system design and interaction. First, we removed the tracking of hands outside the map area to avoid unintended interactions. Second, we improved the accuracy of the pointing position localization while also reducing noise. This was achieved by doubling the size of the index finger position tracking window, correcting minor bugs with the street graphs of our maps, and adding “gravity” to streets, nodes, and points of interest, so that small inadvertent hand movements from the user do not result in leaving the currently indicated feature.

Third, we modified the navigation instructions to be provided one-by-one: when the user asks for directions, the LLM answers with the first instruction only, and the following one is read when the user asks for it. Also, we enabled point of interests to be announced during *point inspection* when they are relevant to the last *verbal query*. Finally, we added the distance information to every navigation step, and we reduced the verbosity of the navigation instructions by removing accessibility indications. These could still be accessed by explicitly asking.

### C. PROMPT AUGMENTATION VERSION 7

To support the new functions, we improved the prompt augmentation as follows. First, the prompt answering instructions were modified to have the LLM provide only the first step of a *navigation instruction*, and to invite the user to ask for the next step in a follow-up request. Also, we added a tool call to make a point of interest discoverable on the map when pointed to. Finally, we updated the map contextual information to distinguish between T and 4-way intersections.

All these changes led to further improvements in benchmark results, with all responses classified as either correct (89.47%) or correct but not optimal (10.53%). Non-optimal responses only were primarily due to verbose answers to questions related to navigation.

## VII. PHASE 4: SECOND REFINEMENT

### A. EVALUATION

The second study adopted the methodology described in Section III-A and was conducted with 3 participants (see Table 3). One of them is blind (**P8**) and two have some residual vision (**P9**, **P10**). **P9** also has reduced touch sensitivity, which impacted some of the study tasks. Indeed the participant did not complete most of the study tasks. Participants had various levels of expertise with braille and LLMs, but all had relatively low expertise with tactile maps. In particular **P9** had prior experience as teachers of BLV students, and was the only participant who had experience with digitally augmented tactile materials, despite a low expertise in tactile maps.

During the evaluation we observed that *MapIO* worked as expected and did not notice any errors in the answers to *verbal queries*. However, we noticed three interaction issues.

First, all participants had difficulties with the voice recording interaction. Very often, **P8** and **P10** would inadvertently touch the laptop’s touchpad while interacting with the space bar, sometimes exiting the system app. Instead, **P9** would press the touchpad instead of the space bar, and had difficulties in pressing the space bar to start recording and press it again to stop. Indeed, the participant would often talk without pressing the space bar, or would press and hold it while talking (a push-to-talk approach [42]). This participant would also often remove hands from the map while asking questions, which resulted in errors when asking questions related to the pointed area.

Second, interaction through *point inspection* felt natural for the participants, but in some cases they interacted differently from what we expected. Specifically, **P9** would keep the hand raised from the map, pointing slightly from above, which was sometimes harder for the system to track. Instead, **P8** would follow the tactile cues with the side of the finger, causing the tracked point to always be a little on the side of the actual tactile element. This participant slightly moved the finger over the tactile cues continuously, triggering repetitive truncated messages due to the movement. **P8** also moved quickly over the intersections and wondered why the system did not completely read the intersections names: *“What is the way to make it say all the intersections?”* After an additional explanation, **P8** learned to hold the pointing finger still on tactile features to enable audio feedback to be read completely without interruption.

Third, all participants had difficulties in following the navigation instructions. In particular, as the turn directions were provided with respect to the route, all participants were confused when their orientation and route orientation differed. For example, when following a route going towards the bottom of the map, a “turn left” instruction means that users should move their finger to the right side of the map. Distance indications were also not useful to the participants as they would perform large movements even when the instruction mentioned very short distances (in particular **P8**). While navigating, **P8** and **P9** would not listen to street and intersection names so they would often miss the turning point mentioned in the instruction, which resulted in faults during navigation. **P9** also asked whether the chime sound (played at the end of the LLM message) meant that the target point has been reached, suggesting that such a cue would be desirable. The difficulty in using the *navigation* functionality elicited alternative strategies from the participants. Specifically, **P9** and **P10** sometimes tried to reach a point of interest by traversing the streets on the map, searching for those mentioned in its address. **P10** also used a form of “beaconing”, iteratively moving and asking for the distance to a point of interest.

### B. FINAL PROTOTYPE

**FIGURE 4.**
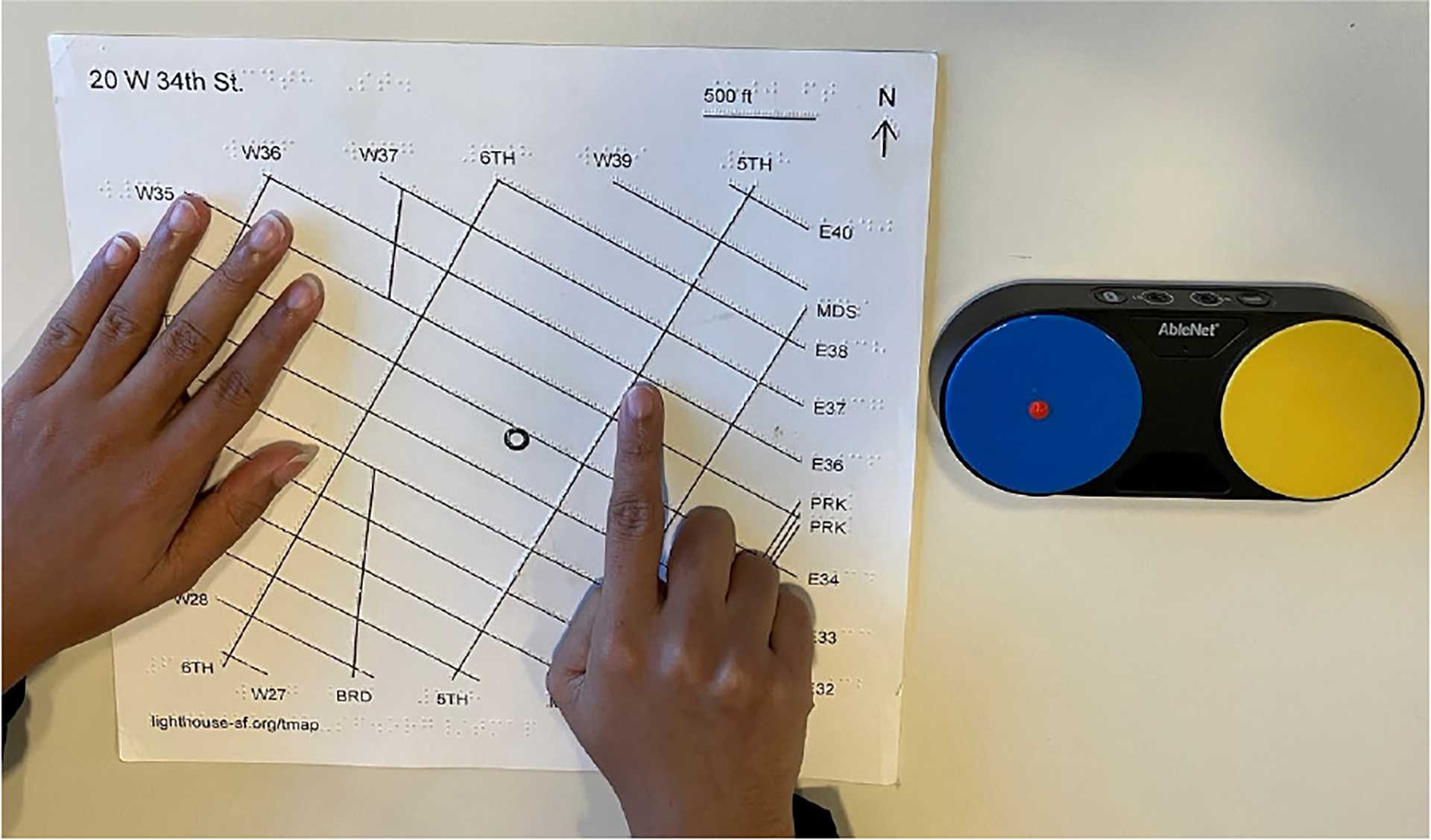
Participant interacting with *MapIO*.

We addressed the issues identified in the second iteration by implementing the following modifications to the system interaction design and prompt augmentations. First, we substituted the keyboard interaction with two physical buttons to avoid accidental pressing of the touchpad or the other keys of the laptop. The final setup is shown in Figure 4. Specifically, one button was defined as the “talk” button, allowing the user to ask questions to the LLM, while the other was used as the “halt” button to silence the system in case of long LLM answers. Also, we changed the speech recording interaction so that the users would need to keep the button pressed while recording their query.

In the pointing interaction, we relaxed the constraints for the recognition of the pointing gesture to better detect when the user is pointing vertically, and we increased the “gravity” parameter of the map features to mitigate problems due to the low accuracy in the detection of the pointing target. We also added the automatic reading of accessibility information if the user keeps pointing to a street or an intersection, as a way to avoid explicitly asking the LLM. We enabled the possibility to point with both hands as well. When two pointing hands are tracked, the system refers to the hand that is currently moving, ignoring the other one. This approach allows a common interaction modality that BLV people use with tactile maps: to “anchor” a point on the map with one hand, and explore with the other, thus allowing to assess relative positions of map features with respect to the anchored point [43].

Concerning the navigation, since asking for the instructions one at a time was still difficult for the participants, we introduced a real time *navigation* functionality called “street-by-street navigation”. This functionality automatically provides the next navigation instruction, once the user completes a navigation step. If the user moves in the wrong direction during a step, the system notifies the user and recalculates the navigation from the new position. The instructions also give distance indications in terms of the number of traversed blocks, and we modified the directions provided by the *navigation* to always refer to the cardinal directions of the map, making them fixed with respect to the user’s reference frame (*i.e*., west would always be to the left of the user). An important aspect of this function is that it is implemented locally by *MapIO* thanks to an additional tool call. This guarantees a more responsive interaction because the LLM initiates the *navigation* only but is not involved in the subsequent navigation interactions.

Finally, we also introduced another *navigation* functionality, called “fly-me-there navigation”, that guides the user to reach a point on the map without following the street graph. This *navigation* modality provides verbal indications of the cardinal direction (north, south, east or west) toward which the user should move the finger to minimize the distance to the target (a beaconing approach). This functionality was meant to be quicker for reaching a target position when it is not needed to know which path to take towards it. This function was implemented with the same approach as the street-by-street navigation so it is run locally by *MapIO*.

### C. PROMPT AUGMENTATION VERSION 8

To support the two new *navigation* modalities, we added two new tool calls, hence adding their definition to the answering instructions as well as the text required to explain to the LLM when to activate one or the other. For instance, if the user asks “guide me to …”, fly-me-there navigation should be activated, while, if they ask “navigate me to …” or “give me directions to …”, street-by-street navigation should be activated. When the context of the question is unclear, the LLM should directly ask the user which option they prefer. This expected behavior is explained to the LLM through example questions paired with their expected answers, which are included in the answering instructions to encourage standardized responses to the most frequent queries. In the eighth version, the benchmark results achieved a score of 94.74% correct responses and 5.26% correct but not optimal on the New York City map.

## VIII. PHASE 5: FINAL EVALUATION

### A. EVALUATION DESIGN

The final evaluation was based on the methodology described in Section III-A with the addition of a final interview stage. The interview aimed at assessing the system usability, collecting feedback about system functionalities, and general reflections on the system’s strengths and weaknesses. The complete list of questions is reported in Supplementary Material. System usability was assessed using the SUS questionnaire [36], with the adaptation proposed by Brock et al. [11], which modifies the seventh question to read, “I would imagine that most BLV people would learn to use this system very quickly”. This change prompts participants to evaluate the system with its intended audience in mind. We descriptively analyzed the overall score and single items, comparing them to benchmarks available in the literature [44], [45].

The observational study and the interview were analyzed through reflexive thematic analysis [35], with both inductive and deductive approaches. The inductive approach was based on the observational analysis of video and audio recordings of the experiments. Three researchers jointly viewed and coded the recordings of one participant, registering emotional responses, behavioral and interaction patterns, and in particular differences between their natural way of interacting through touch and conversation, with respect to the designed interaction paradigm. Then, the rest of the recordings were randomly split between the three researchers, with each video coded by one primary coder, and codes integrated with additional observations from a secondary coder. During a follow up meeting, codes were reviewed and adjusted. Uncertainty or disagreements among the coders were addressed through reviewing and discussion of the relevant parts of the recordings, until consensus was reached. The deductive approach derived theory-driven themes and codes, starting from the examined research questions, the system and interaction design, and the topics investigated in our final questionnaire. These topics were tightly related to the system functionalities and the proposed interaction modality. The codes were finally merged and reviewed by the researchers, grouping them into subthemes and themes.

### B. PARTICIPANTS

The study involved ten participants, whose demographic data are reported in Table 5. The inclusion criterion for this study required the participants to be blind or legally blind. The exclusion criteria were: having familiarity with the area represented in the Detroit map, having participated in the focus group or the previous studies, or the presence of other impairments that could influence the study. Familiarity with the area represented in the New York map was not considered an exclusion criteria, as the map was only used for training purposes.

Seven participants self-identified as female, while the remaining participants identified as male. Participants’ ages ranged from 30 to 78 (56.11 ± 15.73).^13^ Notably, **P12**, **P15**, and **P20** also indicated they had prior experience in training other BLV individuals. Finally, we inquired about participants’ past experiences with digitally augmented tactile materials. **P12**, **P14**, **P16**, and **P20** had little prior experience with digitally augmented tactile materials, primarily during previous experiments at The Smith-Kettlewell Eye Research Institute.

All participants completed the experiment as expected, with an experiment duration of about one hour. All participants were able to complete all the tasks in the formal evaluation, with the following exceptions: in task 8, **P12** misunderstood the question and asked for the wrong point of interest (a hotel, instead of a restaurant). Also, **P15** and **P16** were not able to complete one task (saving a point of interest as a bookmark). **P17** requested not to record the audio. The results of the final questionnaire and SUS with **P15** were lost due to technical problems.

### C. THEMATIC ANALYSIS

Through deductive process we identified 4 main themes, related to the *System* and its functionalities (*point inspection*, *verbal queries* and *navigation*). The inductive process, instead, lead to the identification of 5 themes related to: subjective *Perception* of system aspects or functionalities, their *Use Cases*, the *Naturalness* of the interactions, *Desiderata* regarding existing or new functionalities, and how users address *Fault and Recovery*.

We reflected on the fact that the two categorizations, stemming respectively from the inductive and the deductive approaches, are mutually orthogonal. Discussing how to present the themes, we have realized that presenting the deductive themes as main themes and for each having the inductive themes as sub-themes supports a clearer presentation of the findings. Thus, our presentation follows this rationale.

#### 1) SYSTEM

##### Perception.

Many participants expressed appreciation for *MapIO* (**P12**, **P13**, **P14**, **P16**, **P18**, **P19**, **P20**). In particular, **P14** was impressed by the capabilities of the system and **P20** reported:

“Would have liked to have done more! Want to use the system more!”

##### Use Cases.

Some criticisms were reported as well: **P12**, **P13** and **P16** commented that the system would not be usable on the go while **P19** and **P20** raised the problem of map creation, both in terms of cost and dependence on others to make the maps. Despite this, all participants reported at least one use case in which they would use *MapIO*, including exploration (**P11**, **P12**, **P17**, **P18**), navigation (**P13**, **P14**, **P16**, **P19**, **P20**) and retrieval of accessibility features (**P11**). For instance, **P12** would use *MapIO* to preview an unfamiliar environment.

##### Naturalness.

All participants explored maps with an open hand, some also with two (**P11**, **P13**, **P15**, **P17**, **P18**, **P19**, **P20**). In particular, **P17** explored systematically starting from the map perimeter and from the center. This interaction appears to be natural and it is one of the advantages of the system with respect to approaches using touch surfaces that react to all touches. However, **P20** notes that it does take time to learn to use the system.

##### Desiderata.

System’s ability to be used in diverse contexts, in particular in mobility, was requested by some (**P13**, **P14**, **P17**), while others suggested integration with more portable form factors such as smart glasses (**P16**) or link with other navigation tools such as BlindSquare (**P12**). **P19** foresees possible generalization to other types of tactile materials (*e.g*., diagrams in education). Supporting personalization based on user preferences and interests was also suggested by **P12** (*e.g*., to tag favorite places) and by **P16** (*e.g*., to change distance units).

##### Fault and Recovery.

Despite the appreciation for the system, trust in provided information is one concern that BLV people have when interacting with technology [49]. Indeed, **P19** raised the issue of wanting to cross-check the information provided by the system (*e.g*., using Google Maps).

#### 2) POINT INSPECTION

##### Perception.

All participants appreciated the *point inspection* functionality, and most praised its accuracy (**P11**, **P13**, **P14**, **P16**, **P17**, **P19**). Similarly, all highlighted the clarity of the provided information. In particular the first use of the functionality triggered a strong emotional response in **P11**.

##### Use Cases.

All participants reported the usefulness of the information provided, which was regarded to be complete by most (**P11**, **P14**, **P16**, **P18**, **P19**). However, **P13** highlighted that real world use is needed to assess this aspect, while **P12**, **P16**, and **P20** were similarly impressed by the detail of the provided feedback. Specifically, **P12** commented:

“They are giving terrain information as well! That’s interesting.”

In contrast, some found the information redundant (**P11**, **P18**) or sometimes redundant (**P12**, **P19**). In particular **P18** felt overwhelmed by all the provided information.

##### Naturalness.

During training, the pointing gesture took some time to learn by many participants (**P11**, **P12**, **P16**, **P17**, **P20**), or was not perceived as natural (**P13**, **P15**, **P18**). However, most had no problems with it once learned (as highlighted by **P12**), with the exception of **P20** who had some problems with it also during testing. Once learned, participants would naturally use the pointing gesture to explore alternatively to the open hand exploration (**P12**) or in combination with it (**P15**, **P17**). Concerning the two-hand pointing, **P13** expressed appreciation for it. However, most participants did not use this functionality in practice (**P11**, **P12**, **P13**, **P14**). Indeed **P18** found it confusing, while **P14**, **P15**, **P20** had difficulties when they tried using it. However, **P20** did eventually use this functionality spontaneously.

##### Desiderata.

In addition to using the pointing functionality for accessing street information, some participants (**P11**, **P13**, **P14**, **P15**, **P16**, **P19**) pointed to braille labels, expecting to receive their description. This suggests that the system should also be able to describe braille labels. Despite the appreciation for pointing feedback, in some occasions, participants were annoyed by it (*e.g*., when talking to the experimenter), hinting that pausing audio feedback (**P12**, **P13**, **P16**, **P18**, **P20**) should be a functionality (note that the “halt” function was designed to stop the LLM response only). Some also wanted to turn off the “crickets” sound or to personalize it (**P11**, **P16**).

##### Fault and Recovery.

The “Crickets” sound was nonetheless found to be a welcome indicator of the functioning of the system (**P16**):

“At least crickets tell you it’s still on. If an app is quiet, I don’t know what to do. SeeingAI gives feedback but sometimes it’s silent for a while, now what do I do?”

Similarly the repetitions of audio information were appreciated when information is missed the first time (**P17**). The pointing itself was a potential source of faults as the participants had no clue regarding the area framed by the camera which, if wrongly positioned, could cause issues that participants could not address autonomously (**P12**, **P15**). Occlusion caused by participants’ own body (e.g., one hand in front of the other) was also a potential source of pointing recognition errors, which was hard for the participants to understand (**P11**).

#### 3) VERBAL QUERIES

##### Perception.

Many participants appreciated the *verbal queries* functionality and its ability to understand their questions (**P11**, **P12**, **P13**, **P14**, **P16**, **P17**, **P20**). In particular **P11** expressed enthusiasm when first interacting through conversation. In general, all participants considered the provided answers clear, complete, correct, concise, and useful. Concise responses are also the reason why all participants except **P15** and **P19** never used the halt button to interrupt a response. However, the system could take time to respond to user questions, which could cause annoyance, as expressed by **P19**, or could overwhelm the user by providing too much information (**P12**).

##### Use Cases.

Participants commented on some use cases for which the conversational functionality was considered useful. These include learning about restaurants (**P14**), entertainment options (**P13**) and other points of interest (**P19**) near the pointed position. Even just knowing that a point of interest is on the map is useful (**P12**), and getting the distance and walking time between two points of interest is appreciated as well (**P13**, **P17**). Finally, the ability to remember points of interest as bookmarks was also appreciated (**P11**, **P16**, **P17**, **P18**). In particular, **P17** said:

“I don’t have to find the address again and again. Really loved that!”

##### Naturalness.

For some participants, the interaction with the physical button was not natural. Indeed, some started talking without pushing the button (**P11**, **P13**, **P14**, **P15**, **P16**, **P19**). Others pressed the button but did not keep it pushed (**P12**, **P13**, **P15**, **P16**, **P19**, **P20**). Others again took some time to learn coordinating the pointing hand and the hand pushing the button (**P14**). Also, the physical buttons we used for the experiment were too sensitive and most participants had problems keeping it pressed. This challenge was unexpected and required some training (**P13**, **P14**, **P15**, **P16**, **P17**, **P19**, **P20**). After some practice in the use of the physical buttons most participants could use *verbal queries* without issues. In particular **P12** noted that it is easy to be understood by the system despite phrasing errors (see *Fault and Recovery*). However, some participants forgot how to ask for direction (**P15**) or how to save a bookmark (**P13**). Also, initially **P15** asked incomplete questions (*e.g*., sentences without verb). Similarly, **P19** had to think carefully about the phrasing and **P18** commented:

“It took a bit of practice to figure out how to make myself sound clear to it”

##### Desiderata.

Participants requested various additional information to be made available through conversational interaction. Some requested details about accessibility (**P13**, **P17**, **P19**), others about points of interest and facilities (**P14**, **P15**, **P20**) and others about public transportation (**P12**, **P13**, **P17**).

##### Fault and Recovery.

In most of the cases, if the participant’s request was not correctly understood by the conversational interface, the problem was solved by reformulating the question (**P11**, **P13**, **P16**, **P18**, **P19**). Participants appreciated when the LLM recovered from minor pronunciation or formulation mistakes (**P11**, **P12**, **P14**, **P15**, **P19**, **P20**). For example, **P15** was surprised that the LLM could correctly understand a prompt that he had the feeling was not clearly formulated:

“Very hip. Especially since I mess with your wording. It doesn’t seem to care. It gets the essence of what I’m driving at.”

One exception was with the requests to remember the bookmarks which, if not phrased correctly, were interpreted by the LLM as attempts to store personal information and therefore were not processed (**P15**, **P19**). We highlight that, since *MapIO* uses an LLM for conversational support, hallucinations are also possible. This however is mitigated by our prompt augmentations methodology. Indeed we observed a single case of hallucination, when the system failed to report nearby points of interest (**P15**).

#### 4) NAVIGATION

##### Perception.

Some participants were thrilled when first using *navigation* functionalities (**P11** and **P20** for fly-me-there, **P13**, **P14**, **P20** for street-by-street). However, these functionalities were often hard to use, causing frustration (**P11** for fly-me-there, **P12**, **P14**, **P15**, **P20** for street-by-street). Despite this, most participants managed to complete the navigation tasks, after some learning, as highlighted by **P15**.

##### Use Cases.

Specific use cases for the two *navigation* modalities were clear to the participants: street-by-street is preferable for learning routes (**P13**, **P14**, **P16**, **P17**, **P18**, **P20**), while fly-me-there gives a general direction of a point of interest (**P12**, **P13**, **P16**) or when getting to a location by car or transportation (**P17**).

##### Naturalness.

While street-by-street was generally more useful, it was also more difficult (**P11**, **P14**, **P18**, **P20**). The differences between the two interactions sometimes confused the participants that followed the streets in fly-me-there (**P11**, **P12**, **P14**, **P16**, **P17**,**P18**, **P19**, **P20**), or went over them in street-by-street navigation. Another interaction problem was due to the fact that some participants were not proficient with cardinal directions, in particular the diagonal ones (*e.g*., “North-east”) that were common in the New York City map (**P11**, **P12**, **P13**, **P14**, **P16**, **P19**, **P20**). In several cases the participants moved beyond the destination point during navigation due to a system delay in updating the position (**P11**, **P13**, **P14**, **P16**, **P17 P18**, **P19**). This also resulted in too many messages being provided (**P15**).

##### Desiderata.

The system delay (**P13**, **P16**, **P19**) and generally the reliability during navigation (**P14**, **P20**) were also the main requests for improvement for this functionality. Furthermore, to address the lack of proficiency with cardinal directions, **P11** proposes to use clockface directions.

##### Fault and Recovery.

System delays would cause some participants to start moving erratically due to frustration (**P13**, **P16**, **P17**, **P20**). This situation was often addressed by slowing down (**P11**, **P14**, **P16**, **P17**) or by backtracking (**P16**). In sporadic cases, during navigation, there were problems in computing the homography due to the fact that users were covering a large part of the map with their hands. This in turn resulted in position computation errors, ultimately preventing the user from finding the destination (**P19**).

### D. USABILITY EVALUATION

**FIGURE 5.**
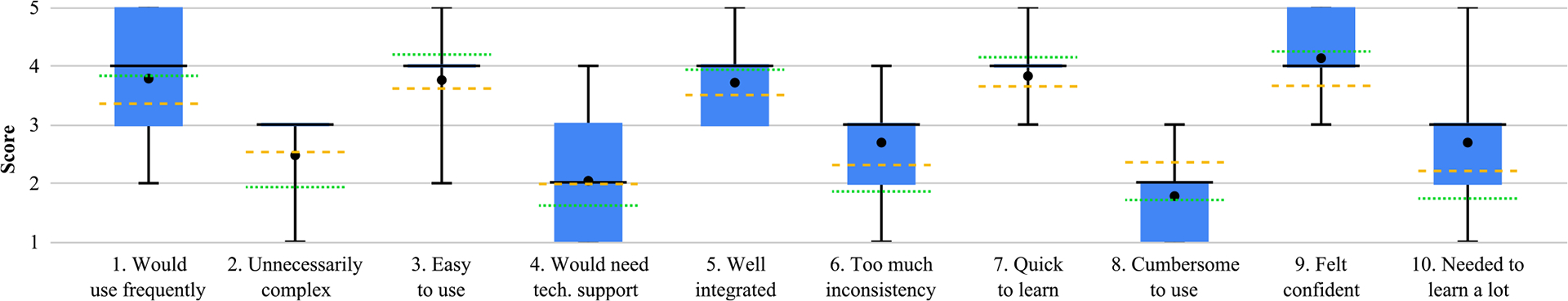
SUS item results, aggregated over participants (• Mean, – Median, - - Benchmark Average, … Benchmark Good).

System usability was generally rated positively (see Figure 5), with an average score of 68.61±15.16. Based on benchmarks of previously collected SUS data, this result is regarded as “good” [44]. Considering the specific questionnaire items, in relation to prior benchmarks [45], we note that most questions score “above average”. Specifically the participants were eager to use the system frequently (**Q1**, 3.89 ± 1.05), did not find it unnecessarily complex (**Q2**, 2.67±0.71), and found it easy to use (**Q3**, 3.78±0.83). They did not feel the need for technical support (**Q4**, 2.11±1.27), and perceived the functionalities to be well integrated (**Q5**, 3.78 ± 0.83). Participants did not perceive the system to be cumbersome (**Q8** 1.78 ± 0.83), and they felt confident using it (**Q9**, 4.22±0.67). However, while they found the system to be quick to learn (**Q7**, 3.88±0.60), they felt that they needed to learn a lot of things before using it (**Q10**, 2.78±1.20). Also, they perceived the system to be inconsistent (**Q6**, 2.78±0.97). We suspect this is due to the navigation issues outlined above.

The results show differences based on participants’ prior experience with tactile maps. Participants with low experience reported a lower overall usability score (58.33 ± 14.22) compared to those with medium to high experience (73.75 ± 13.85). Looking at individual questionnaire items, less experienced users found the system less intuitive and more difficult to use overall. Specifically, they reported that they would use the system less frequently (**Q1**, 2.67±0.58 for less experienced users vs. 4.50 ± 0.55 for users with higher experience), that they found it harder to use (**Q3**, 3.33 ± 1.15 vs. 4.00 ± 0.63) and that they felt less confident while using it (**Q9**, 3.67 ± 0.58 vs. 4.50 ± 0.55). Participants with low experience perceived system functionalities to be less well integrated (**Q5**, 3.00 ± 0.00 vs. 4.17 ± 0.75) and more inconsistent in their behavior (**Q6**, 3.33 ± 1.15 vs. 2.50 ± 0.84). The system was also more cumbersome to use for them (**Q8**, 2.00±1.00 vs. 1.67±0.82). The learning curve was perceived as steeper (**Q7**, 3.67±0.58 vs. 4.00±0.63), and less experienced users felt they needed to learn more before using it (**Q10**, 3.33 ± 1.53 vs. 2.50 ± 1.05). However, they also reported that they would need less technical support than more experienced users (**Q4**, 1.67 ± 1.15 vs. 2.33 ± 1.37). Both groups gave similar scores regarding the complexity of the system (**Q2**, 2.67 ± 0.58 vs. 2.67 ± 0.82). Due to the relatively small sample size (3 users with low experience with tactile maps, 7 with medium to high experience), we were not able to determine if these differences are statistically significant.

## IX. DISCUSSION

We discuss the key results of our study, highlighting the way we designed the system and summarizing the types of interactions that BLV participants engaged in with our LLM-enabled DATM. In addition, we address the main limitations of our methodological approach.

### A. SYSTEM DESIGN

The system was designed iteratively in multiple stages. This cycle of iterations allowed to identify the use cases (**RQ1**) and to ensure that the development of new functionalities was heavily influenced by observations of BLV participants, with the functionality being iteratively debugged and improved after live participant experiments.

A key to the capability of the final system was the augmentation of the LLM with specific tool calls. In the initial iterations of the system, we had expected that the LLM could handle most functionality, aside from modules such as computer vision, speech-to-text and text-to-speech. Over time, however, the iterative process of prompt augmentation engineering demonstrated the inability of LLM models to reliably answer spatial queries (**RQ2**), a problem that was addressed by refining the contextual information provided to the LLM and by incorporating multiple tool calls to ensure reliable spatial reasoning (**RQ2.1**). While the reliance on tool calls limits the generalizability of the solution, we anticipate that the spatial reasoning abilities of LLMs will become more robust in the future, reducing or eliminating the need for tool calls.

### B. OBSERVED INTERACTIONS

BLV participants in the final evaluation interacted with *MapIO* with a combination of gestures and vocal interactions (**RQ3**). They perceived most system functionalities as useful and clear (**RQ4**), despite some initial difficulties using *MapIO*. Indeed, a substantial amount of training was required to accomplish this. This is partly due to the need for non-visual explanations, *e.g*., how to hold the finger to make a pointing gesture and how to operate the Bluetooth button to ask a question, which tend to require more verbalization (and time) than the types of visual explanations that sighted individuals typically incorporate (*e.g*., a photo of the desired pointing gesture).

Most participants found the pointing functionality useful and accurate, even if it sometimes triggered announcements with too much information. After training, participants were able to make the pointing gesture reliably. Problems occasionally arose in pointing gesture recognition, mostly when the pointing finger was not held sufficiently parallel to the map surface, causing perspective foreshortening in the camera’s view of the finger. Since conducting the study, the authors have devised a new version of the pointing gesture recognition algorithm that addresses this problem, which will be incorporated in the future.

Many participants appreciated the ability to converse with the LLM and the opportunity to ask a wide range of questions, as well as the ability to bookmark points of interest for later reference. However, the over-sensitivity of the Bluetooth button used to record the voice caused some confusion. Delays in the LLM response were also inconvenient, especially when the response was lengthy. While long answers negatively affected the delay, they were not perceived as too long by the participants (who used the halt button in only a few occasions) with the only exception of the list of navigation instructions provided by *MapIO* in the initial prototype (see Section VI-A).

We observe that different participants had different ways to respond to navigation instructions, as previously observed in the literature for egocentric navigation assistance [50]. Indeed, participants found the fly-me-there and street-by-street navigation functions the most difficult part of the system, but most were able to master these functions after enough practice, and most acknowledged the value of receiving navigation instructions to a point of interest. The challenges were caused by a few factors, including (a) confusion about the meanings of the cardinal directions (north, west, southeast, etc.) on the physical DATM and (b) feedback delays, which forced participants to move their pointing finger slowly and often resulted in announcements that were not timely.

These challenges will be mitigated in the future by allowing the user to choose to receive directions in different formats (such as left, down, or 2 ‘oclock) and by using a faster system (see Sec. IX-C). Another factor that possibly influenced the usability of the two distinct *navigation* functionalities is that their need emerged in the second observational study and hence, differently from the other functionalities, they have not been iteratively refined.

Overall perception of *MapIO* was positive, with several participants excited by the access that it provided to many types of information, despite the limitations they noted.

### C. LIMITATIONS

Our final experiment was conducted with 10 BLV participants, and a total of 20 representative users and stakeholders were involved in the system design and development process. While such numbers are in line with the prior accessibility research [51], [52], we realize that a greater number of participants, possibly involved in a longitudinal evaluation process, would be needed to ensure that the proposed approach is widely generalizable.

To enable the participants to use the system during the experiments, a lengthy training session of about 30 minutes was required, which meant that the experimenters had limited time to assess participants’ ability to use the system independently (to prevent the entire session from being too long). A longer assessment would have allowed us to explore how participants used the system to solve a larger variety of tasks. Moreover, our assessment consisted entirely of a set of tasks specified by the experimenters, which limits our ability to observe how participants would use the system in actual use cases. It is also unclear whether users would remember how to use the system after prolonged lack of use.

The interface was constrained by the need to call OpenAI’s GPT-4o for each query, which created unpredictable delays (from under a second to more than 10 seconds). In the future we will explore LLM models that can be run locally on the PC, such as LLama 2 or DeepSeek, which we anticipate will solve this problem. In addition, the limited frame rate of the overall system (roughly 10 frames per second) made it difficult for *MapIO* to keep up with participants’ moving fingers. We believe that system performance can be substantially improved by engineering the software.

At the time that we developed our prototype system, LLM models with integrated voice interactions were not yet available as API services. As a result, we were forced to use speech-to-text and text-to-speech, which resulted in an unnatural interface that required the user to hold down the “talk” button while recording their question. In the future, we expect that LLM models will expose voice interactions through an API, which should allow the user to pose *vocal queries* without the need for a physical gesture to indicate the intention to talk.

### D. SYSTEM SCALABILITY AND REAL-WORLD APPLICATION

The *MapIO* system can be extended to other contexts and domains well beyond the application in this paper, which was implemented on an ad hoc basis for the purposes of our study. For example, it would be possible to adapt the system to work with essentially any tactile map at an urban level. This is supported by our results: we refined the prompt on one map (New York) and then we evaluated it on a different map (Detroit) and we still got almost perfect results. So we believe that the same prompt will be effective for other maps, as well. A different issue related to the adaptation of *MapIO* to other maps is related to the creation of the map contextual information (see Section III-B). This information can be generated with a little manual effort (e.g., to specify the geographic location of the map center, the scale and extent of the map) and with automated tools to extract the road network and point of interest data from existing services like Google Maps or OpenStreetMap. The tool could be also made compatible with existing tools such as TactileImages’ Mapy image editor,^14^ which can generate map models given a street address. In this way *MapIO* could be integrated with a tactile map-printing service to create tactile maps that are supported by the system.

Alternatively, we can make *MapIO* work with existing physical tactile maps (e.g., representing up-to-date geographical data as well as historical or fictional maps), for which no digital model is available. For this purpose, we have already developed a tool that allows us to upload an image of the map, select the nodes and edges of the graph of map data (corresponding to intersections and the streets connecting them) and automatically create the graph in the correct format. The tool could be enhanced by using computer vision and AI (e.g.,^15^) to automatically segment the roads and other paths on the map, after which the user would make any necessary edits or corrections to the resulting segmentation.

We note that the *MapIO* system relies on tool calls to overcome deficiencies in the LLM’s spatial abilities. However, LLMs and other AI models are rapidly evolving and we foresee that eventually few (if any) of these tool calls will be necessary. This implies greater generalizability of the system across multiple problem domains in the future. Examples of these additional problem domains include the following: (a) maps of road networks that are more complex than those typical of the US, such as European road networks with many curved paths, which may demand alternative navigation instructions rather than relying mainly on simple cardinal directions (East/West/etc.); (b) 2D floor plans of buildings, which consist of networks of corridors, rooms and points of interest that can be organized using a graph data structure; and (c) 3D maps [25], which are often easier for blind users to grasp than 2D maps [53], but which require more complex geometric analysis of camera images to determine the pose of the user’s hand and fingers relative to the map.

While our implementation of *MapIO* suffers from latency issues because it runs on a computer that accesses GPT-4o in the cloud, the system can be adapted to run on multiple platforms. First, other LLMs such as Llama, Qwen, Florence and DeepSeek run locally on a laptop, which will greatly reduce latencies. Instead of requiring a hardware button to start and stop the recording of voice commands, these recordings could be triggered simply by the user uttering “Hey MapIO”. At some point we anticipate that sufficiently powerful LLMs will run on mobile platforms, which will enable the entire system to operate on a mobile device (perhaps with a cell phone holder or tripod to mount the mobile camera to acquire a clear view of the map). Finally, we are exploring the possibility of porting *MapIO* to a wearable camera glass platform, such as Meta Ray-Bans, which are becoming increasingly popular among blind individuals [54]. While the system would still likely need to stream video images to a laptop running an LLM, this platform has the great advantage of making it easy for a blind user to aim the Ray-Bans camera at the tactile map [55], simply by orienting their head towards the map (which is much easier than having to aim a handheld or external camera towards a visual target).

### E. ADDRESSING THE NAVIGATION CHALLENGE

In the earlier versions of *MapIO*, we relied on the LLM’s capabilities to interpret the road network and give navigation instructions based on it autonomously. However, this approach led to poor results, as the model struggled to provide accurate directions. To address this issue, in the first prototype of *MapIO* we relied on a tool call that interfaced with Google Routes API to retrieve navigation instructions. While this method improved accuracy, the evaluation of this initial prototype revealed that the responses were too long, as navigation instructions were provided all at once, making them difficult to follow. In the first refinement of the system we therefore switched to a one-by-one approach, in which the user had to ask for the next instruction upon completing the previous step. However, explicitly requesting for the next instruction also resulted in overly long and complex interactions. Ultimately, in the second refinement of *MapIO* we implemented automated detection of the completion of a navigation step, providing instructions for the following one. Specifically, two distinct navigation modes were implemented: street-by-street navigation, which provided detailed turn-by-turn instructions following the street graph, and fly-me-there navigation, which offered direct guidance to the destination coordinates using cardinal directions, without considering the street graph.

Despite these improvements, navigation remained challenging in the latest iteration of our system as well. The thematic analysis revealed that one issue was the confusion between the two navigation modes, as users sometimes struggled to differentiate them and would follow streets when fly-me-there navigation was active or go over them in street-by-street mode. Additionally, some participants demonstrated low proficiency with cardinal directions. Another limitation was the system’s delay in updating the user’s position, causing them to move past their destination and often resulting in an excessive number of unnecessary messages. This lack of reliability led to frustration among participants, highlighting the need for further refinements.

To address these issues, several improvements can be considered. First, to reduce confusion between the two navigation modes, we could introduce distinct auditory cues to clearly differentiate them and provide a brief contextual reminder when a mode is activated — such as a short explanation of how the instructions will be provided — helping users better understand and follow the navigation guidance. Second, to mitigate difficulties with cardinal directions, we could adopt the clock face direction instructions, as suggested by one participant. This approach would not only make navigation more intuitive for users unfamiliar with cardinal directions terminology but also partially address the challenges posed by cities with different street layouts, as discussed in the previous section. Lastly, to minimize delays in position updates, improvements in system engineering are necessary. Optimizing the code to accelerate the recognition of the pointed location and increasing the system’s frame rate could lead to more responsive navigation assistance, reducing errors and unnecessary messages.

## X. CONCLUSION AND FUTURE WORK

This paper describes *MapIO*, a novel system that augments a tactile map with a LLM to create a conversational interface for BLV users. The system adds audio interactivity to the tactile information present in the map, enabling the user to receive immediate text-to-speech announcements about features they point to on the map, and also to verbally ask a wide range of questions to the system. In addition, *navigation* functions give the user audio directions to guide them to a desired point of interest on the map. The system was developed in an iterative process combining feedback from BLV participants, an iterative prompt engineering process to optimize the accuracy and usefulness of the LLM responses, and experiments with BLV participants using the prototype. Tests were conducted with 10 BLV participants using the system, demonstrating their ability to perform a set of tasks relating to a realistic scenario in which the participant is asked to plan an evening with friends in an unfamiliar urban setting. We analyze the type of questions the users are willing to ask and study how the users interact with the system, its usability, and user experience.

In future work, we plan to exploit recent improvements to LLM models that offer integrated voice interactions. We expect that ongoing improvements in LLM spatial reasoning abilities will allow the updated MapIO system to reliably answer an even wider range of questions, with less reliance on ad hoc prompt engineering and tool calls that limit the generalizability of the current system.

## Supplementary Material

supp1-3566286

supp2-3566286

## Figures and Tables

**TABLE 1.** Demographic information on formative questionnaire participants.

PID	Age	Gender	Visual Impairment		TBLV	Experience with TMs
			Description	Onset		
**P1**	46	M	Blindness	birth	Yes	High
**P2**	46	F	Blindness	N/A	No	Mid
**P3**	29	F	Low vision	birth	Yes	Mid
**P4**	43	M	Blindness	birth	Yes	High
**P5**	60	F	None		Yes	High

TBLV: teacher of BLV students, TM: tactile map.

**TABLE 2.** Benchmark results.

V.	Answering instructions	Map contextual information	Output of PCD Generator	Results (%)					
				DW	NRQ	BW	PI	CNO	C
1	Neighborhood resident, conversational, detailed yet concise	Textual description of the map area	Address of the position pointed to	5.26	28.95	2.63	0.00	13.16	50.00
2	As above + rely only on the provided info	As above + list of PoIs, map coordinates and scale	Camera image (map and hand)	18.42	0.00	34.21	0.00	5.26	42.11
3			Synthetic image (map+marker)	21.05	0.00	26.32	0.00	15.79	36.84
4		As above + local reference system + road graph as JSON	Local coord., closest edge, address, distance from nodes	10.53	0.00	10.53	0.00	15.79	63.16
5		As in I4 but road graph as text		2.63	0.00	2.63	0.00	13.16	81.58
6	As above + tool calls for spatial reasoning			0.00	0.00	2.63	0.00	7.89	89.47
7	As above + one-by-one navigation instructions + tool call for activating points of interest	As above + specify if each node is T or 4-ways intersection	As above + type of closer intersection (T or 4 ways)	0.00	0.00	0.00	0.00	10.53	89.47
8	As above + tool call for navigation			0.00	0.00	0.00	0.00	5.26	94.74

V: version, PCD: prompt contextual data, DW: deceptively wrong, NRQ: not replying to question, BW: blatantly wrong, PI: partial or incomplete, CNO: correct but not optimal, C: correct.

**TABLE 3.** Participants in the first study.

PID	Age	Gender	Visual Impairment			TBLV	Expertise		Experience with DATS
			Description	Onset	Residual		Braille	TMs	
**P6**	47	F	Fully blind	birth	No	Yes	High	High	Yes
**P7**	37	M	LCA [39]	birth	LP	Yes	Mid	Low	Yes

LP: light perception, TBLV: teacher of BLV students, TM: tactile map, DATS: digitally augmented tactile supports.

**TABLE 4.** Participants in the second study.

PID	Age	Gender	Visual Impairment			TBLV	Expertise			Experience with DATS
			Description	Onset	Residual		Braille	TMs	LLMs	
**P8**	75	F	Fully blind	birth	No	No	High	Low	High	No
**P9**	51	M	CVI [40]	birth	20/400	Yes	None	Low	Mid	Yes
**P10**	46	M	Optic Atrophy [41]	birth	20/400	No	Mid	Mid	Low	No

TBLV: teacher of BLV students, TM: tactile map, DATS: digitally augmented tactile supports.

**TABLE 5.** Participants in the main study.

PID	Age	Gender	Visual Impairment			TBLV	Expertise			Experience with DATS
			Description	Onset	Residual		Braille	TMs	LLMs	
**P11**	39	F	ROP [46]	birth	20/200	No	High	Low	Low	No
**P12**	68	M	Glaucoma	birth	Yes	Yes	Mid	Low	Mid	Yes
**P13**	52	M	RP [47]	age 7	LP&FP	No	None	Mid	High	Yes
**P14**	63	F	Blindness	age 1	No	No	High	Mid	None	Yes
**P15**	78	M	Blindness	birth	No	Yes	High	Mid	None	Yes
**P16**	43	F	Uveitis [48]	age 5	No	No	None	Mid	High	Yes
**P17**	N/A	F	Genetic mutation	birth	LP	No	Low	Mid	None	No
**P18**	30	F	ROP	birth	LP&FP	No	High	Low	None	No
**P19**	56	F	LCA [39]	birth	LP	No	High	High	High	Yes
**P20**	76	F	ROP	birth	LP&FP	Yes	High	Mid	None	Yes

LP: light perception, FP: form perception, TBLV: teacher of BLV students, TM: tactile map, DATS: digitally augmented tactile supports.

## Footnotes

1
https://lighthouse-sf.org/tactile-images-maps/tmap

2
https://www.sensationalbooks.com/products.html#blackboard

3
https://www.orbitresearch.com/product/graphiti

4
https://www.humanware.com/monarch

5
https://irie-at.com/product/feelif-pro/

6
https://www.touchgraphics.com/store/p/t3-tactile-tablet

7
https://tactileimages.org/en/home

8
https://platform.openai.com/docs/guides/prompt-engineering#tactic-ask-the-model-to-adopt-a-persona

9 We used the ChatGPT-4o-2024–08-06 model, accessed through its API.

10
https://cloud.google.com/speech-to-text

11 We used the pyttsx3 Python library.

12
https://developers.google.com/maps/documentation/routes

13 We use the notation *x* ± *y* to denote average *x* and standard deviation *y*.

14
https://tactileimages.org/en/image-creator

15
https://segment-anything.com
