# Supplementary material for: MapIO: A Gestural and Conversational Interface for Tactile Maps
Source: IEEE Access. Author manuscript; Available in PMC 2025 Aug 29. (PMC12392394; doi:10.1109/access.2025.3566286)
Supplement: supp2-3566286 [file NIHMS2083087-supplement-supp2-3566286.pdf]

MapIO: a Gestural and Conversational Interface for Tactile Maps - Supplementary Materials

MATTEO MANZONI, Università degli Studi di Milano, Italy
SERGIO MASCETTI, Università degli Studi di Milano, Italy
DRAGAN AHMETOVIC, Università degli Studi di Milano, Italy
RYAN CRABB, The Smith-Kettlewell Eye Research Institute, USA
JAMES M. COUGHLAN, The Smith-Kettlewell Eye Research Institute, USA

A Formative questionnaire

1. What is your age? Please specify in years.
2. What is your gender?
   a. Male
   b. Female
   c. Non binary
   d. Prefer not to answer
   e. Other
3. Do you have a visual disability? If so, please specify what type of visual disability, onset age and if you have residual sight.
4. Do you have professional experience training BVI individuals, e.g., as a Teacher of the Visually Impaired (TVI) or Orientation & Mobility (O&M) specialist?
5. How much experience do you have with tactile maps?
6. Consider a smart tactile map of a city that combines a traditional tactile map with a computer application that can read aloud the names of the roads when you point to them with your finger. You can also verbally ask a question regarding the map content and the application will read aloud the answer. When you ask questions, you could also point to a location on the map with your index finger and the question will refer to that point. Could you give us some examples of questions that you would ask to such a system? If appropriate, specify whether you would also point to a target location while asking the question.
7. What might you use this system for?

B Benchmark questions

Table 1. Questions included in the benchmark to evaluate prompt augmentation strategies

Question	Repetitions
Is there a T intersection here?	Repeated 3 times: at a T intersection, at a four-way intersection, and in the middle of a street
Tell me the roads parallel to this one.	Repeated 3 times; in the middle of a west-east street, in the middle of a north-south street, and at an intersection

Authors' Contact Information: Matteo Manzoni, Università degli Studi di Milano, Milano, Italy, matteo.manzoni2@studenti.unimi.it; Sergio Mascetti, Università degli Studi di Milano, Milano, Italy, sergio.mascetti@unimi.it; Dragan Ahmetovic, Università degli Studi di Milano, Milano, Italy, dragan.ahmetovic@unimi.it; Ryan Crabb, The Smith-Kettlewell Eye Research Institute, San Francisco, CA, USA, ryan.crabb@ski.org; James M. Coughlan, The Smith-Kettlewell Eye Research Institute, San Francisco, CA, USA, coughlan@ski.org.

Question	Repetitions
Will <POI> be open an hour from now?	Repeated 3 times: during the POI's regular opening hours, after its closing hours, and on weekends within its weekly schedule
Is a TM of the <POI> available inside the building?	
Is there a walk light here?	Repeated 3 times: at an intersection with a walk light, at an intersection without walk lights, and in the middle of a street
Tell me if there are stairs at some point on the road where I am right now.	Repeated 2 times: on a street with stairs on the way, and on a street without stairs
Tell me the roads intersecting this one.	Repeated 2 times: in the middle of a street, and at an intersection
Will I make it to <POI> before it closes?	Repeated 2 times near the POI's closing time: from a location very close to the POI, and from a distant position
Considering the route that goes from <POI 1> to <POI 2> passing through <street 1> and <street 2>, which restaurants can I find along the route.	
How many streets are there on the map?	
What Italian restaurant are there on the map?	
What's the scale of the map?	
Tell me more about this street.	
Tell me the characteristics of the road surface of the street where I am right now.	
Tell me the name of this street.	Repeated 3 times: on two different street, and at an intersection
Where am I?	Repeated 2 times: in the middle of a street, and at an intersection
How can I come here from <POI>?	
How can I get to <POI>?	
How far is <POI> on foot?	
Considering the route that goes from <POI 1> to passing through <POI 2> and <POI 3>, which accessibility features should I be aware of?	
Describe this block	
Give me an overall description of the map.	
What is there around my current position?	
What are the main landmarks in the area?	

C Prompt augmentation versions 1 to 5

In the following we report the details of the iterative prompt augmentation refinement from version 1 to version 5.

C.1 First version

In the first version we relied solely on pre-existing LLM knowledge to generate responses. The answering instructions specified to the LLM that the user is blind and gave it the role of a long-time resident of the neighborhood. This was intended to encourage the LLM to provide details and create a friendly, conversational tone. Additionally, answering instructions specified how to react to ambiguous or unclear queries - by asking for clarification - and to respond directly, with a detailed yet concise answer. The map contextual information included only a brief textual description of the map, saying "I am in New York, in the Empire State Building district". Meanwhile, the prompt contextual data generator was designed to compute a textual description of the user's position and current time, like, for example, "at the intersection between Broadway and West 35th Street" followed by "The current time is: ...".

The goal of this version was to explore the extent of the knowledge of the LLM. The benchmark revealed a 50.00% rate of correct responses, with most correct answers concerning well-known points of interest, such as the Empire State Building, or notable roads like Broadway. When the LLM encountered queries about less relevant points of interest (e.g., Cooper Electrics, West 38th Street, New York City), it often acknowledged the lack of information (28.95% of responses). However, in some instances, it generated fabricated responses (hallucination): 5.26% of responses were classified as deceptively wrong, and 2.63% as blatantly wrong. These findings highlighted the inadequacy of this approach (**Rq2**) and convinced us to explore different forms of map contextual data and policies for the prompt contextual data generator.

C.2 Versions 2 and 3

Since the first version revealed the limitations of the LLM's knowledge, we aimed to enhance its performance in the second and third versions by including in the map contextual data a list of points of interest that includes shops and other public places extracted from a public source[1]. This list was presented to the LLM in textual form, reporting, for each point of interest its location (in terms of latitude and longitude) and descriptive information, like name, category, extended description, opening hours, accessibility, etc. We also provide a reference system: the four map corners and the scale ("The scale of the map is: 60m: 1cm"). The answering instructions were also expanded to prevent the LLM from accessing its pre-existing knowledge by saying "Stick to the provided information: when information is insufficient to answer a question, respond by acknowledging the lack of an answer and suggest a way for me to find one."

The prompt contextual data generator varied across the two versions. In the second version, it produced the RGB image acquired from the camera. This image frames the map and the user hand that is aiming the target of the map. Instead, in the third version, the prompt contextual data generator processes the camera image by using a combination of algorithms based on machine learning and rules, and computes the pointed position. Then, it augments a pre-existing image of the map with a marker representing the user's position and returns the result.

We expected these changes to improve the performance, instead benchmark results showed only 41.11% of correct answer for the second version and 36.84% for the third, less than in the first version. Similarly, the percentage of responses classified as deceptively wrong and blatantly wrong increased, rising to 18.42% and 34.21% for the second version, and 21.05% and 26.32% for the third. Our interpretation of these results is that the LLM is unable to perform spatial reasoning based on the provided images. This is also reported in the OpenAI documentation[2].

[1] apidocs.geoapify.com
[2] https://platform.openai.com/docs/guides/vision#limitations

C.3 Versions 4 and 5

In order to address the issues that arose in versions 2 and 3, we extended the map contextual information to explicitly represent the map's road network as a graph, where nodes indicates intersections between streets and edges represent street segments. This representation allows us to include information about the accessibility of nodes and edges, such as the presence of crosswalk signals at intersections or ongoing roadwork on specific street segments. Additional contextual data about the map, including its name and nearby locations (north, east, south, and west), are provided to the LLM. We also introduced a local Euclidean reference system. Nodes are placed within this system in such a way that the distance between any two nodes matches the real-world distance, in meters, between the corresponding intersections. A point of interest's position is represented in multiple formats: in terms of the local reference system, the address, and the closest edge on the graph. The intuition is that, while in principle the latter two formats can be derived from the local position, the conversion can be subject to errors by the LLM. With the proposed solution the LLM can select the format that is more practical for a given task, hence improving the quality of the answer. In the fourth version, the graph was provided to the LLM in a JSON format along with a final brief explanation of its structure. In the fifth version, it was provided as text, where graph elements were alternated with their descriptions to create a more cohesive description.

Similarly to a point of interest's position, the pointing position is also reported in various formats in addition to the local reference system, including the closest edge, the edge name and the distance from the edge nodes as in "[...] the closest point on the road network is on edge n1 - n2, which is part of West 38th Street, between 5th Avenue and 6th Avenue. I'm at a distance of 100 m from the intersection with 6th Avenue and 210 m from the the intersection with 5th Avenue."

Benchmark results showed the best results so far. Both versions achieved more correct answers than the previous ones: 63.16% and 81.58% respectively. The text-based representation (Version 5) yielded particularly good performance, with only one answer evaluated as deceptively wrong and one as blatantly wrong. However, the LLM continued to struggle with some questions that requires spatial reasoning, for example, "How can I come here from Solle Spa?" and "How far is Solle Spa on foot?". In the former, the LLM completed the answer with wrong accessibility information, while in the latter, it slightly miscalculated the distance.

D Experimental protocol

D.1 Inclusion criteria

Participants must be blind or legally blind and should have no familiarity with the Conant Gardens district of Detroit, Michigan. Familiarity with the Empire State Building area of New York, whose map is used during training in the study, is not considered an exclusion criteria.

D.2 Demographic questionnaire

1. What is your age? Please specify in years.
2. What is your gender?
   a. Male
   b. Female
   c. Non binary
   d. Prefer not to answer
   e. Other
3. What visual disability do you have? Please specify type of visual disability, onset age and if you have residual sight.
4. Do you have any light perception or form perception?

5. Do you have professional experience training BVI individuals, e.g., as a Teacher of the Visually Impaired (TVI) or Orientation & Mobility (O&M) specialist?
6. Do you read braille? If so, would you rate your level or reading ability as low, medium or high? specialist?
7. How much experience do you have with tactile maps?
   a. I don't know what tactile maps are
   b. I never used tactile maps
   c. I used tactile maps a few times in my life
   d. I use tactile maps from time to time
   e. I regularly use tactile maps
8. Are you very familiar with the city of Detroit, Michigan?
   8.1. If so, are you very familiar with the Conant Gardens district of Detroit?
9. How much familiarity do you have with LLM chatbots (eg. ChatGPT)?
   a. I don't know what LLM chatbots are
   b. I never used LLM chatbots
   c. I used LLM chatbots a few times in my life
   d. I use LLM chatbots from time to time
   e. I regularly use LLM chatbots
10. Did you ever experience "smart" tactile materials that also provide interactive verbal or auditory feedback? If so, what were they about? Were they more understandable or interesting? In general, what positive and what negative aspects can you report about this experience?

D.3 Introduction to the system

> You will be working with a tactile map of a neighborhood of New York City, and then another tactile map of a neighborhood of Detroit. At the beginning, the system will read aloud a map description and initial instructions. Then you can get information from the system in two main ways: you can point to features and hear pointing feedback about them, or else you can use the question and answer mode to ask questions and hear answers. With the pointing feedback, as you explore the map, you can point your index finger to any street or intersection, and the system will respond with its name and main characteristics, like the road surface or its slope. With the question and answer mode you can ask questions aloud. Questions can be about points of interest in the area, such as shops, tourist attractions, restaurants, cafes, and banks. Additionally, you can request directions or accessibility features in the neighborhood. During the experiment, feel free to say what you are thinking about your interaction with the system. This "think-aloud" process will help the experimenters understand how you interact with the system. Also, feel free to ask any questions to the experimenters.

Once the system is activated:

> Please let us know if the volume needs to be lower or higher. Make sure you can clearly hear the text-to-speech, "ding" sound and crickets sound.

D.4 Training phase

The training phase uses a map of the Empire State Building neighborhood, in New York City, and it is divided in pointing training (Subsection D.4.1), question and answer training (Subsection D.4.2), and directions training (Subsection D.4.3). Note that some of the points of interest on the map are fictional and created specifically for the experiments.

D.4.1 Pointing training.

Now we will explain how you can point to features on the map to activate “pointing feedback”, which reads the road names and provides details for roads and intersections.

Task 1:

To use the “pointing feedback”, hold your index finger outstretched with your other fingers curled in. (This is a “number one” gesture, which is a fist with just the index finger pointing straight and out.) Your entire index finger and hand should be visible to the camera mounted above the map; to be as clearly visible as possible, your index finger should be nearly parallel to the map, not pointed straight down perpendicular to the map. The best way to do this is to keep your entire hand, not just the fingertip, touching the map surface and not raise up. If you have long fingernails, the system may slightly mis-estimate where your fingertip is touching the map, so please keep this in mind.

The system will ignore your hands if you don’t make this pointing gesture. In fact, you can explore the map with your fingers outstretched for one or both hands, and the system will ignore your hands during this time.
The system will make a ding sound when it sees you are making a new pointing gesture. Whenever a pointing gesture is not visible (either because the hand isn’t visible or it is visible but it’s not making a pointing gesture), the system plays a cricket sound.

For practice, we will use a map of New York City. Explore the map with your open hand (which won’t trigger any pointing feedback). Start by finding the edges of the map. You will also find a small circle in the middle of the map indicating the map center. Then point to a road so that its name will be read aloud. [max 2 minutes]

Task 2:

In addition to reading aloud road names as soon as you point to them, you can also get detailed information about roads and intersections by standing still with the finger for a moment.

Please practice this: get the details about at least one road and one intersection. Then trace the road along its entire length on the map with your fingertip. [max 4 minutes]

Task 3:

If the system sees two pointing gestures at the same time, it will track the location of the index finger that has most recently moved. That way you can keep one finger anchored in a location, which is a good way to avoid losing track of where that location is.

Please practice this: anchor one hand to a location and then use the other hand to explore the map. Be sure to use the pointing gesture on both hands. [max 2 minutes]

D.4.2 Question and answer training.

Now we will explain how to interact with the system using the “question and answer mode”.

We now give you two external buttons. The one on the left has a small bump dot on it and is the “talk” button. The one on the right (without the bump dot) is the “halt” button. Please position the buttons where it is more convenient for you, but not too close to the map. You can change the button position at any time. The supervisor will help you position the buttons where they are not framed by the camera.

Task 1:

If you want to ask the system a question, you can press and hold the “talk” button and then say your question aloud. Do this as a “tap and hold” with one finger. When you’ve finished asking your question, wait a second, then release the button and the system will respond to your question. If you release the button too early, the last part of your question will not be recognized. The system will

process your question, which will take anywhere from a split second to 10 or more seconds. During this processing period and while the response is being played back, pointing feedback is temporarily disabled. Once the system is done playing its response, the ding sound is played again, and then pointing feedback is enabled again.

Please practice. Press the button and ask a question, like “Is there a Korean restaurant on the map?”. Feel free to ask other questions. [max 2 mins]

Task 2:

You can also ask questions while pointing to a location on the map. In this case, the question can be related to the pointed position.

Please practice. Use the pointing gesture to point to a location on the map and then, while holding your finger there, ask questions related to that point, like “which (or where) is the closest restaurant?” Feel free to ask other questions. [max 2 mins]

Task 3:

In some cases, the answer can be long and have details you are not interested in. You can interrupt the answer by pressing the “halt” button.

Please practice. Ask the system “what are the restaurants in the area?” and halt the answer before it ends. [max 1 min]

D.4.3 Directions training.

Now I’ll describe how you can ask for directions from the system. When you’re pointing to a specific location, you can ask the system for directions to any destination on the map, such as another intersection, a specific restaurant (such as “the nearest Italian restaurant”), etc. The system can guide you with the "fly-me-there guidance" or the "street-by-street navigation".

Task 1:

"Fly-me-there guidance" will take you to the destination, disregarding the street layout. This is useful, for example, if you just want to find a point on the map. With the "Fly-me-there guidance" the system will give you compass directions that you will need to follow. North is toward the top of the map, south toward bottom, west on the left, and east to the right. Far north, far east, etc. means you need to move at least 4 inches to get to the destination. Once you get to the destination, the system plays a ping sound and informs you that the destination is reached.

Practice this. Point a location on the top left part of the map and ask the system “guide me to the Bank of America”. Feel free to practice with other points of interest, like the closest Italian restaurant. [max 3 mins]

Task 2:

The other form to provide directions is called “street-by-street navigation” which provides you instructions to reach your destination along the roads. Starting from your current position, the system reads the direction of the street to follow and for how many blocks. The direction will be either north, south, east, west, northeast, southeast, southwest or northwest. Move your fingertip slowly in the indicated direction! Once you reach the intersection where you need to turn, a confirmation sound will be played (it sounds like "ka chunk"). Stand still for about two seconds and the system will give the next direction. Once you get to the destination, the system plays a ping sound and informs you that the destination is reached. If you get lost, simply stand still for a moment and the system will recompute the route.

Please practice. Find the Bank of America using the "street-by-street directions" by asking “navigate me to the Bank of America”. [max 5 mins]

Task 3:

While you are asking questions, the system can detect that you are interested in some points of interest that will be enabled, which means that, once you touch them on the map, the system plays a ping sound. If your fingertip stands still on that point, the name and details will be provided. You can also give names to points of interest.

Practice this. Ask the system where are the Chinese restaurants. The system reads their names aloud and also enables them. Choose one of the Chinese restaurants and find it, either with fly-me-there guidance or street-by-street directions. Once you find it, ask the system to remember it as your favorite Chinese restaurant. [max 5 mins]

Next, move away from your favorite Chinese restaurant. Then ask the system “navigate me to my favorite Chinese restaurant”. Follow the instructions to reach the restaurant. Feel free to practice with other destinations. [max 5 mins]

Quick review: If you ask to “guide me” to a place, the system gives fly-me-there guidance. If you ask to “navigate me” to a place, the system gives street-by-street directions.

D.5 Formal evaluation phase

The evaluation phase uses a map of the Conant Gardens district of Detroit and is organized into ten tasks, each of which the user must complete within a certain time limit. In this phase, the supervisor is not permitted to assist participants in task completion. However, if the user fails to complete a task and a subsequent task depends on its successful completion, the supervisor shows the participant how to complete it. Note that some points of interest on the map, such as the Sheraton Commander Grand Lake hotel, are fictional and were created specifically for the experiments. In particular, the Sheraton Command Grand Lake hotel was given a long name to make it harder to remember and to encourage users to ask the system to bookmark it with a different name.

You are visiting Detroit and need to organize a night out with friends at a restaurant. Your task is to use the tactile map of a neighborhood in Detroit to explore the neighborhood and identify suitable restaurants.

We will give you a maximum time limit for each of ten tasks. You can ask as many questions as you want to the system.

Task 1 Explore the map freely for one minute, and use the question and answer mode to ask something. [max 2 minutes]

Task 2 You will be staying at the Sheraton Commander Grand Lake hotel. Is this hotel on the map? [max 2 minutes]

Task 3 Is there free Wi-Fi at the hotel? [max 2 minutes]

Task 4 Since the hotel has a long name, ask the system to remember it as “my hotel”. [max 2 minutes]

Task 5 You want to find a nice restaurant where you can meet your friends for a late evening dinner. Check if there is an Italian restaurant. [max 2 minutes]

Task 6 Check if the restaurant is open at 10 PM. [max 2 minutes]

Task 7 Check how long it would take you to walk there from the hotel. [max 2 minutes]

Task 8 You decide to go by taxi. Ask the system to help you point to the restaurant on the map. Once your finger is pointing there, please keep it pointing there for the next two tasks. [max 3 minutes]

Task 9 You want to check what entertainment options are near the restaurant. [max 2 minutes]

Task 10 After the dinner you want to go back to the hotel. Ask the system to navigate you there. [max 5 minutes]

D.6 System usability scale

The System Usability Scale (SUS) questionnaire follows the adaptation proposed by Brock et al., which modifies the seventh question to read, "I would imagine that most BLV people would learn to use this system very quickly". This change prompts participants to evaluate the system with its intended audience in mind.

1. I think that I would like to use this system frequently.
2. I found the system unnecessarily complex.
3. I thought the system was easy to use.
4. I think that I would need the support of a technical person to be able to use this system.
5. I found the various functions in this system were well integrated.
6. I thought there was too much inconsistency in this system.
7. I would imagine that most visually impaired people would learn to use this system very quickly.
8. I found the system very awkward to use.
9. I felt very confident using the system.
10. I needed to learn a lot of things before I could get going with this system.

D.7 Final questionnaire

1. How well did the system recognize your pointing gesture?
2. How well did the system recognize when you were pointing to a specific map feature, e.g., a street or an intersection, to get immediate feedback (without asking a verbal question)?
3. When you pointed to a map feature (without asking a verbal question), what was the quality of the feedback in terms of (a) clarity, (b) usefulness and (c) completeness? Was any information provided by the system unnecessary or redundant?
4. Judging by how the system responded to your verbal questions, how well did it seem to understand your questions? Was it easy to make yourself understood?
5. When you asked verbal questions of the system, what was the quality of the responses in terms of (a) clarity, (b) usefulness, (c) correctness and (d) completeness? (e) Was any information provided by the system unnecessary or redundant?
6. What kind of useful information did the system give you that goes beyond the information contained in the tactile map itself? (Did you use any of the braille symbols on the map?)
7. The system was trained to provide information about the road network, points of interest and accessibility features. Do you think that any relevant information is missing?
8. The system provided two ways to guide you to a destination: navigation by following a route street-by-street, and "fly me there" which help you pinpoint it on the map, without following the roads. How clear and useful do you think they are for their respective tasks? Which way did you prefer and why?
9. If you had this system, what sorts of tasks might you use it for?
10. What was good about the system overall?
11. What was poor about the system overall?
12. What improvements does the system need overall?
13. Do you have any other feedback about the system or today's experiment?
